# Completion of the 5′ end of the porcine teschovirus genome reveals a functional S-segment and enables reverse genetics

**DOI:** 10.1128/jvi.01036-26

**Published:** 2026-08-11

**Authors:** Sebastian Affeldt, Sandra Barth, Stephanie Schlimbach, Irmin Lobedank, Josef Kuehling, Thomas Timm, Günter Lochnit, Sabrina Becker, Enrica Sozzi, Sven Reiche, Friedemann Weber, Gerald Reiner, Benjamin Lamp

**Affiliations:** 1 Institute of Virology, Justus-Liebig-Universität Gießen 9175, Gießen, Germany; 2 Department for Veterinary Clinical Sciences, Justus Liebig University Giessen 9175, Giessen, Germany; 3 Institute for Biochemistry, Justus Liebig University of Gießen, Gießen, Germany; 4 Istituto Zooprofilattico Sperimentale della Lombardia e dell'Emilia Romagna "Bruno Ubertini", Brescia, Italy; 5 Department of Experimental Animal Facilities and Biorisk Management, Friedrich-Loeffler-Institut, Greifswald, Germany; The University of Texas Southwestern Medical Center, Dallas, Texas, USA

**Keywords:** porcine teschovirus, 5’-UTR, S-segment, reverse genetics, infectious cDNA clone, reporter virus, replicon, leader protein, leaderless PTV

## Abstract

Porcine teschoviruses (PTVs) are swine-specific picornaviruses associated with neurological and systemic disease, ranging from severe paralytic Teschen disease to milder forms of porcine encephalomyelitis, known as Talfan disease. Despite their global distribution and veterinary relevance, complete genomic information and molecular tools for PTVs are not available. Here, we report the first complete genome sequences of PTV-A11 (strain Dresden) and a recent PTV-A field isolate (strain Gi2020_1). Both genomes, each approximately 7.2 kb in size, include a previously uncharacterized 5′-terminal region upstream of the poly(C) tract (S-segment), thereby elucidating the complete genomic architecture of PTVs for the first time. The conserved 117–118 nucleotide S-segment closes a long-standing gap in teschovirus genomics and is essential for viral replication. Using reverse genetics, we established infectious molecular clones of both strains that recapitulate the properties of their parental strains. These systems enabled functional analyses of viral gene products, including the demonstration that the leader protein is dispensable for genome replication and morphogenesis but contributes to efficient virus growth. In addition, we developed a replication-competent subgenomic replicon and engineered fluorescent reporter viruses, including a stable mCherry-expressing virus that supports robust infection analysis and allows quantitative protein expression measurements. Together, these findings define the complete PTV genome organization and provide a versatile molecular toolbox for studying teschovirus replication, pathogenesis, and control.

**IMPORTANCE:**

Teschen disease was once a devastating neurological disease of swine caused by highly virulent porcine teschovirus strains (PTVs) but has become rare following their eradication. Current control strategies base on hygiene measures and herd-specific vaccination providing limited protection and leaving swine populations vulnerable to the (re-)emergence of neuroinvasive PTVs. Less virulent strains remain endemic worldwide and continue to impair animal health, welfare, and production efficiency. Progress toward broadly effective vaccines and antivirals has been constrained by incomplete knowledge of the PTV genome. Here, we identify and functionally define the previously unrecognized S-segment that completes the 5′ UTR of PTVs. We further established infectious cDNA clones and developed subgenomic replicons, leaderless viruses, and fluorescent reporter viruses. Our tools enable direct genetic manipulation of PTVs. They provide a platform for mechanistic studies of viral replication, attenuation, and antigen design supporting rational development of vaccines and antiviral strategies against emerging PTVs.

## INTRODUCTION

**Fig 1:**
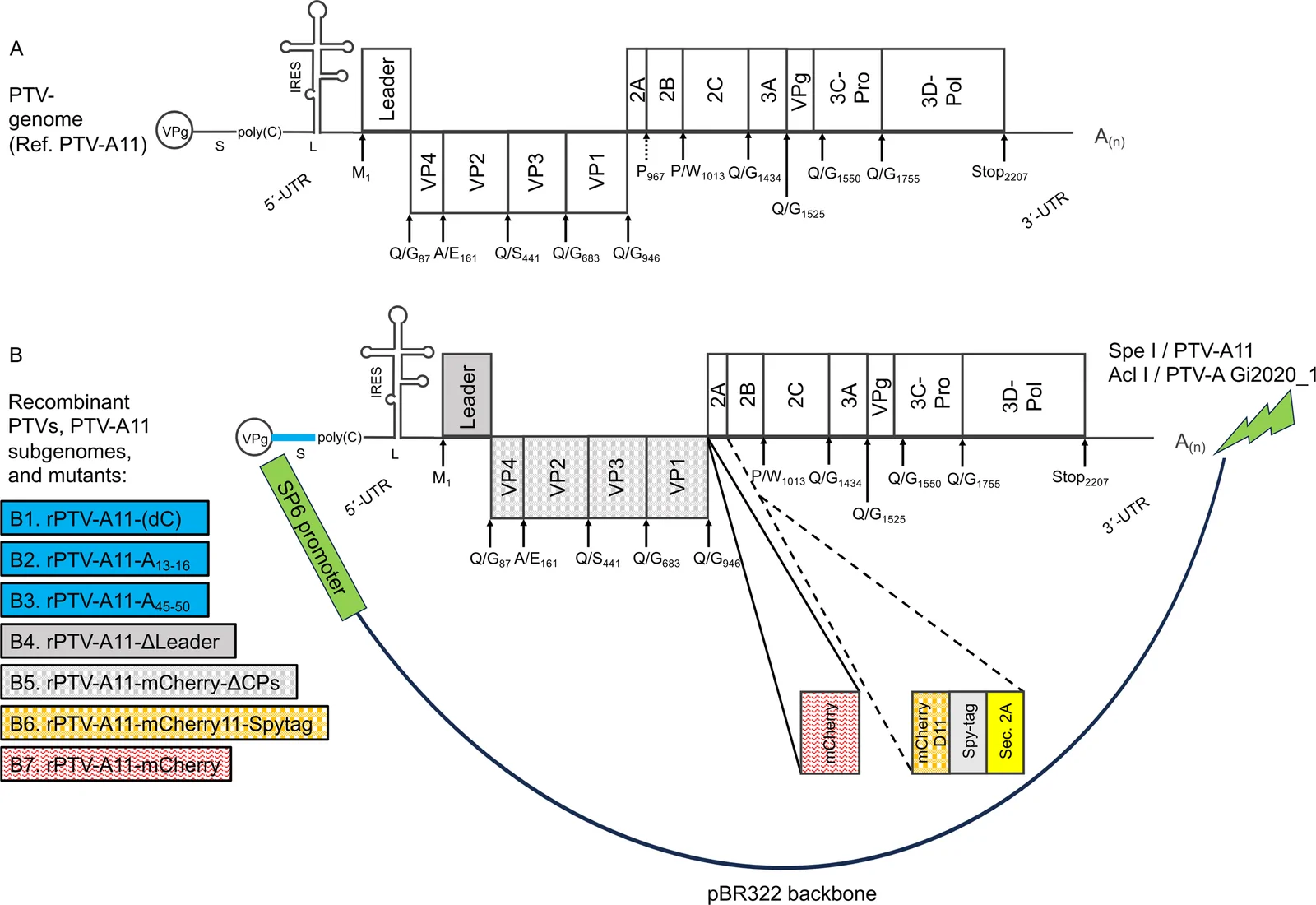
PTV genome organization and recombinant constructs. (**A**) Schematic representation of the PTV genome (PTV-A11, strain Dresden). A genome-linked protein (VPg) is attached to the 5′-end. The 5′-UTR contains the S-segment (S), the poly(C) tract, and the L-segment (L) including the IRES. The ORF encodes a leader protein (Leader), the structural protein region (VP4-VP1), and the nonstructural proteins (2A, 2B, 2C, 3A, VPg, 3C, and 3D). The short 3′-UTR and poly(A) tail [A_(n)_] are indicated. Polyprotein cleavage sites are shown with their respective P1 and P1′ residues in PTV-A11. (**B**) Recombinant viral genomes generated in this study. Full-length cDNAs of two PTV-A strains were cloned into a pBR322 backbone containing an upstream SP6 promoter and a downstream restriction site for linearization (SpeI for PTV-A11 and AclI for PTV-A Gi2020_1). Derivatives based on the PTV-A11 cDNA clone include a nonreplicative genome lacking the 5′-terminal 118 nucleotides upstream of the poly(C) tract (B1), two S-segment mutants (B2 and B3), a leaderless genome (B4), a replication-competent subgenomic replicon expressing mCherry in place of the VP region (B5), a split-mCherry reporter virus (B6), and a full-length mCherry reporter virus (B7).

Teschoviruses are small, non-enveloped, positive-sense RNA viruses that infect pigs and belong to the *Picornaviridae* family (1, 2). Originally classified as porcine enteroviruses (PEVs) (3, 4), they were reassigned following taxonomic revision by the International Committee on Taxonomy of Viruses into the genus *Teschovirus* (5–7). Within this new genus, two novel species were established, namely *Teschovirus asilesi* (PTV-A) and *T. bishikawa* (PTV-B) (8, 9). The former PEV serotypes were renamed into PTV-A1 to PTV-A13 and PTV-B1 to PTV-B3 (10–12). The remaining PEV serotypes were re-classified as two novel species termed *Sapelovirus anglia* grouped in the new genus *Sapelovirus* (PSV-A, formally PEV 8) and *Enterovirus geswini* within the genus *Enterovirus* (EVG1 and EVG2; formally PEV 9 and 10) (13). Genetic recombination plays a key role in the evolution of PTVs and has also been detected between the two species PTV-A and -B (14, 15). PTVs exhibit genome organization and polyprotein processing strategies typical of picornaviruses. A viral protein is covalently linked to the 5′ end of the RNA (VPg). The genome consists of a long 5′ untranslated region (UTR), a single open reading frame (ORF), and a short 3′-UTR including a poly(A) tail (Fig. 1A). The PTV ORF encodes a Leader protein, four structural proteins (VP1-4), and the typical non-structural protein cassettes (2A-C and 3A-D). Owing to its high variability, VP1 is commonly used for genotyping (11). In picornaviruses, the 5′-UTR contains functional independent, cis-acting RNA elements that are pivotal for replication and viral translation, including the internal ribosome entry site (IRES) that mediates cap-independent translation (16). Point mutations within the 5′-UTR can markedly alter viral phenotypes, affecting, for example, the plaque morphology in cell culture, temperature sensitivity of replication, or viral (neuro)virulence (17). In several picornavirus genera, two RNA segments (S and L) are separated by a homopolymeric cytosine sequence [poly(C) tract]. A poly(C) tract was first identified in the encephalomyocarditis virus (EMCV, *Cardiovirus rueckerti*) (18) and subsequently found in other cardioviruses as well as in the foot-and-mouth disease virus (FMDV, *Aphthovirus vesiculae*). Typically, the 3′-terminal regions of the poly(C) tracts contain interruptions by other nucleotides, most commonly uridines (19). Although the exact function of the poly(C) region has not yet been fully elucidated, its shortening or deletion reduces virulence in the natural host without impairing viral replication *in vitro*, suggesting a role in evading the host’s innate immune response (20, 21).

The poly(C) tract separates a shorter upstream UTR segment (S-segment) from a larger downstream segment (L-segment) (22). These two segments are not only separated but also functionally distinct and essential for RNA replication (S-segment) or RNA translation (L-segment), respectively (23). The S-segment comprises approximately 150 nucleotides in EMCV and about 360 nucleotides in FMDV (24, 25). Secondary RNA structures have been identified within the S-segments that resemble those found at the 5′ termini of other picornaviruses (26). For example, the poliovirus (PV, *Enterovirus coxsackiepol*) genome forms a highly stable cloverleaf structure (5′-CL), characterized by extensive G/C base pairing. This cis-acting structure is essential for RNA stability, negative-RNA-strand synthesis, and overall replication. Current models assume long-range interactions between the 5′-CL, an internal cis-replication element (cre) and the poly(A) at the 3′ end of the viral RNA with 3CD. These interactions are pivotal for the formation of a circular, replicative nucleoprotein complex (27). Notably, the 5′-CL is required for the uridylation of the VPg, which then hybridizes with the poly(A) at the 3′ end of the genome initiating negative strand synthesis (28). Similar to the 5′-CL, the S-segment of the FMDV 5′-UTR folds into a long hairpin structure that is indispensable for viral replication and contributes to the modulation of the host innate immune response (29). Even minor mutations within the 5′ terminus of the genome can abolish viral viability if they disrupt these critical secondary structures (30). In contrast, the IRES within the L-segment directly interacts with components of the host translational machinery allowing synthesis of viral proteins under conditions where cap-dependent translation is suppressed as part of the antiviral response (31–33). The IRES of teschoviruses has been functionally characterized and classified as type IV (hepatitis C virus-like) (34, 35). However, the 5′-terminal S-segment sequence of the PTV has not yet been cloned or characterized, limiting a full understanding of genome organization and replication mechanisms (34).

Some selected genera of the family *Picornaviridae* within the subfamilies *Caphthovirinae*, *Kodimesavirinae*, and *Ensavirinae* encode a Leader protein (36). These Leader proteins are highly diverse and represent distinct strategies for host modulation. In aphthoviruses, the Leader protein is a papain-like cysteine protease that mediates viral polyprotein processing and induces host translation shutoff through cleavage of eIF4G (37). In addition, the FMDV Leader functions as a potent antagonist of innate immunity by targeting key components of the interferon induction cascade, including NF-κB and interferon regulatory factors (38). The cardiovirus Leader protein lacks protease activity and instead acts as a multifunctional regulator of host signaling. These proteins disrupt nucleocytoplasmic trafficking and reprogram host cell kinase pathways, thereby preventing the activation of antiviral transcription factors and suppressing the interferon response (39–41). The Leader proteins represent classical virulence factors that, in many cases, are not essential for viral replication *in vitro* but are essential for efficient replication and virulence in the natural host (42). The function of the teschovirus Leader protein has not yet been elucidated. Current evidence suggests that it also lacks protease activity and is cleaved by the viral 3C protease at a conserved cleavage site. However, there is no mechanistic understanding of its role in the virus’s life cycle or of its interactions with the host. Given the central role of Leader proteins in host shutoff and immune evasion across different picornaviruses, we hypothesize that the teschovirus Leader protein exhibits functional convergence.

Classical Teschen disease was caused by the highly virulent and neurotropic PTV strain Teschen (Serotype 1; now PTV-A1). The Teschen strain emerged in 1929 in Teschen (today the Polish-Czech twin city of Cieszyn/Český Těšín) causing polioencephalomyelitis epidemics in swine with high morbidity and mortality (43, 44). Vaccination campaigns followed by a rigorous stamping-out policy resulted in the eradication of the Teschen strain in Europe in the 1970s (45). Yet, outbreaks of highly virulent, neuroinvasive PTV-A1 strains are still observed in other parts of the world, with outbreaks of Teschen disease most recently occurring in 2002 in Canada (46) and in Haiti in 2009 (47). Less virulent PTVs are associated with milder disease forms commonly referred to as Talfan disease, named after its first appearance in the hills of Wales (5, 48, 49). Talfan disease is characterized by low morbidity and mortality rates, typically does not lead to complete paralysis and mainly affects younger pigs. It is still frequently reported from all over the world (46, 49–51). PTV infections have been linked to a variety of clinical symptoms including reproductive disorders, intestinal diseases, pneumonia, pericarditis, and myocarditis and are suspected of synergizing with other agents (52–54). Although most circulating strains are of low virulence and endemic in pig populations worldwide, sporadic outbreaks of highly virulent variants underscore the need for improved molecular understanding and control strategies (11, 12, 55).

The aim of this work was to advance the molecular characterization of teschoviruses and to develop experimental tools for their study. Following the identification of the previously unresolved 5′-terminal S-segment, we determined the first complete genome sequences of two teschovirus strains. Establishing reverse genetics for the prototype PTV-A11 strain Dresden and a recent field isolate (PTV-A Gi2020_1), we confirmed the integrity and functionality of these genomes. In addition, we generated replicative subgenomes, reporter viruses, and a leaderless PTV. This toolbox provides a foundation for future studies on teschovirus biology and will facilitate the rational development of antiviral strategies and vaccines.

## MATERIALS AND METHODS

### Cell culture and viruses

PTVs can be propagated in various porcine cell lines, including SK-6, PK-15, ST, and STE cells (55). In this study, we used SK-6 cells (56), which were maintained in Dulbecco’s modified Eagle’s medium (DMEM) supplemented with 10% fetal calf serum (FCS Gold; Bio&Sell, Feucht, Germany) at 37°C with 5% CO_2_. For virus propagation, SK-6 cells were infected with the PTV inoculum for 1 h, after which the medium was replaced. Virus-containing supernatants were harvested at 72 h post infection (hpi) or upon complete lysis, as described earlier (57). Supernatants were clarified by filtration through a 0.45 µm cellulose syringe filter (Minisart; Sartorius, Göttingen, Germany), aliquoted, and stored at −80°C. Virus titers were determined as 50% tissue culture infectious doses (TCID_50_) by endpoint dilution assays in triplicates based on cytopathic effect and subsequent immunofluorescence tests. Titers were calculated using the Spearman-Kärber method (58). Statistical analyses were based on three independent biological experiments. Differences were considered statistically significant at *P* < 0.05. For statistical analyses, the virus titers were log₁₀-transformed. Differences in progeny virus production were evaluated at 72 hpi by one-way analysis of variance (ANOVA) followed by Tukey’s multiple-comparison test using GraphPad Prism (GraphPad Software). The PTV-A11 strain Dresden (AF296096.1, Ref-SKU 015V-01826) and the field isolate PTV-A Gi2020_1 (MW824925) were obtained from the reference strain collection of the Institute of Virology, Justus Liebig University Giessen, or isolated from a recent outbreak of porcine encephalomyelitis in Germany (to be described elsewhere). To assess the stability of recombinant viruses, 5 × 10^6^ SK-6 cells were electroporated with *in vitro* transcribed RNA (see below) and seeded in 6-well plates. Supernatants were harvested at 72 hpi (passage 0, P0) and 100 µL were used to infect naïve SK-6 cells in 6-well plates for P1. The P1 supernatant was again harvested 72 hpi and used to infect a fresh 6-well plate (P2). This procedure was repeated for 10 passages. For the low-titer construct (PTV-A11-mCherry11-Spytag), 1 mL of supernatant was used per passage. In parallel, transfected cells of PTV-A11-mCherry11-Spytag were passaged at a 1:10 ratio for 10 passages to facilitate adaptation and selection of variants with improved replication. After 10 passages, the titer, phenotype, and sequence of the viruses were analyzed following plaque purification. For plaque purification, the viruses were titrated in multiple replicates on naive cells in 96-well plates, and a well was selected in which only a single viral plaque was detectable in the last virus-positive dilution step. The supernatant from these cells was then used to infect new cells. Plaque purification was always performed twice to ensure that clonal viral genomes with the highest titer were selected.

### Indirect immunofluorescence assays and Western blotting

For indirect immunofluorescence assays, cells were fixed with 4% paraformaldehyde for 20 min at 4°C and permeabilized with 0.5% (vol/vol) Triton X-100 (Merck, Darmstadt, Germany) in phosphate-buffered saline (PBS). Primary monoclonal antibodies (MAbs) or sera were applied as indicated. Phosphate-buffered saline containing 0.05% Tween-20 (PBSt) was used for washing and antibody dilution. Cy3- or FITC-conjugated goat anti rabbit IgG (111-165-144; Jackson ImmunoResearch, Ely, Cambridgeshire, United Kingdom) or conjugated goat anti mouse IgG (115-165-003, Jackson ImmunoResearch) were used for the detection of primary antibody binding applying an inverted fluorescence microscope (Olympus IX70, Shinjuku, Japan) or a digital fluorescence microscope (EVOS M3000; Thermo Fisher, Waltham, MA, USA). For intensity analyses, fluorescence microscopy images of virus-infected cells were analyzed using Fiji (x86-64) based on ImageJ (59). Images were imported in their original format and converted to 8-bit grayscale without additional preprocessing. Intensity profiles were generated by drawing a diagonal line region of interest (ROI) across each image using the line selection tool. Fluorescence intensity along the selected ROI was quantified using the “Plot Profile” function, yielding a one-dimensional intensity distribution (*x*-axis: pixel position; *y*-axis: signal intensity). The resulting intensity values were exported as numerical data and visualized using Microsoft Excel. Selected intensity profiles with the corresponding fluorescence images are provided in the supplemental material.

For Western blotting, cells were directly harvested in SDS-PAGE loading buffer and heated at 95°C for 5 min. Proteins were separated in 7.5% (wt/vol) polyacrylamide tricine gels and transferred onto nitrocellulose membranes (Pall, Pensacola, FL, USA). The membranes were blocked with 5% (wt/vol) skim milk (Carl Roth, Karlsruhe, Germany) in PBSt. MAbs and sera were used as indicated and detected by peroxidase-labeled goat anti-mouse IgG (115-035-003; Jackson ImmunoResearch) or goat anti-rabbit IgG (111-035-003; Jackson ImmunoResearch). ECL-Prime substrate was applied as chemiluminescence reagent (GE Healthcare, Chicago, Illinois, USA), and photon emission was recorded via an imaging system (Odyssey M; LICOR, Lincoln, NE, USA).

### Antibodies and antisera

A neutralizing rabbit antiserum specific for PTV-A11 was provided by the Istituto Zooprofilattico Sperimentale della Lombardia e dell'Emilia Romagna “Bruno Ubertini” (Brescia, Italy). Murine monoclonal antibodies (MAbs) 4B1 (Ref-SKU: 015A-01734) (60) and 1B9 (Ref-SKU: 015A-01733) (46), which are cross-reactive with multiple PTV strains, as well as the PTV-A11-specific MAb 3C3 (Ref-SKU: 015A-01745), were obtained from the Friedrich-Loeffler-Institute (Riems, Germany). The anti-mCherry MAb (clone OTI10G6; TA180028; OriGene, Rockville, MD, USA) and the anti-dsRNA MAb (clone J2; MABE1134; Sigma-Aldrich, St. Louis, MO, USA) were purchased commercially.

### Two-dimensional gel electrophoresis and MALDI-TOF mass spectrometry

For two-dimensional (2D) gel electrophoresis, protein samples were solubilized in denaturing buffer (6 M urea, 2 M thiourea, 4% CHAPS, 1% DTT, and 2% Pharmalyte 3–10). Proteins were separated in the first dimension by isoelectric focusing using immobilized pH gradient strips (IPG pH 3–10) to a total of 32.05 kVh. Following focusing, IPG strips were equilibrated in buffer (6 M urea, 50 mM Tris-HCl [pH 6.8], 30% glycerol, 0.01% bromophenol blue, 0.1 mM EDTA) supplemented with SDS and DTT, followed by a second equilibration step in the same buffer supplemented with SDS and iodoacetamide. Proteins were then resolved in the second dimension by SDS-PAGE as described above and either stained or transferred to PVDF membranes.

For matrix-assisted laser desorption ionization-time of flight mass spectrometry (MALDI-TOF MS), proteins and excised protein spots from PVDF membranes were reduced, carbamidomethylated, and digested with trypsin. Peptide masses were analyzed using an Ultraflex TOF/TOF mass spectrometer (Bruker Daltonics, Bremen, Germany) equipped with a nitrogen laser, LIFT-MS/MS, and Compass v1.4 software including FlexControl v3.4, FlexAnalysis v3.4 and BioTools v3.2. The instrument was operated in positive-ion reflectron mode with a matrix comprising 2,5-dihydroxybenzoic acid and methylendiphosphonic acid. Proteins were identified by peptide mass fingerprinting using MASCOT (Matrix Science, Boston, MA, USA) against the NCBInr and UniProt databases, as well as a custom database derived from the translated ORF of the PTV-A Gi2020_1 genome. Search parameters included a mass tolerance of 75 ppm, fixed carbamidomethylation of cysteine residues, and variable oxidation of methionine. A false discovery rate of 5% was applied.

### PTV preparation

Virus-containing supernatants were harvested at 48–72 hpi and clarified by centrifugation at 2,500 × *g* for 15 min at 4°C. Virions were pelleted through a 25% sucrose cushion by ultracentrifugation at 35,000 rpm for 3 h (Type 45 Ti rotor; Beckman Coulter, IN, USA). Pellets were resuspended in phosphate-buffered saline (PBS) containing 0.1% Triton X-100 and incubated for 24 h with gentle agitation. The suspension was clarified by centrifugation at 15,000 × *g* for 1 min at 4°C and subjected to cesium chloride (CsCl) density gradient ultracentrifugation. Separation was performed using a continuous CsCl gradient (0–1.5 g/mL) and centrifugation at 35,000 rpm for 20 h at 4°C (SW41 Ti rotor; Beckman Coulter). Fractions were collected, assessed using ELISA, and analyzed for infectivity by endpoint dilution on SK-6 cells. Based on this distribution of antigen and infectivity, virions were subsequently purified using a step gradient consisting of 1.25 g/mL CsCl layered over a 1.40 g/mL CsCl cushion. Virus particles accumulated at the interface and were collected, followed by buffer exchange to PBS using a Capto Core 400 column (HiTrap 1 mL; GE Healthcare).

### Non-infectious cDNA clone of PTV-A11

Total RNA was isolated from PTV-A11-infected SK-6 cells at 24 hpi using the QIAamp Viral RNA Mini Kit on a QIAcube platform (Qiagen, Hilden, Germany). Based on the published genome sequence of PTV-A11 strain Dresden (AF296096) (7), viral cDNA was generated by one-step RT-PCR using the OneTaq One-Step RT-PCR kit (New England Biolabs, Ipswich, MA, USA) with primers PTV-5′-Oligo(dC)_forw and (dT) (Table 1). The resulting 7.15 kb amplicon was integrated into a pBR322-derived vector containing an SP6 promoter and a unique SpeI restriction site for linearization prior to *in vitro* transcription. The vector backbone was amplified using Q5 polymerase (NEB) and primers PTV-pBR-(dA)_forw and PTV-pBR-(dG)_rev to introduce complementary overhangs. Insert and vector fragments were purified (Monarch DNA Gel Extraction Kit; NEB) and assembled using NEBuilder HiFi DNA Assembly (NEB) yielding plasmid PTV-A11-(dC)_pBR (Fig. 1B). All constructs were verified by long-read sequencing (Full PlasmidSeq; Microsynth, Balgach, Switzerland). For RNA transcription, plasmid DNA was linearized with SpeI, purified, and transcribed *in vitro* using SP6 RNA polymerase. Transcripts were electroporated into SK-6 cells as described in “Genomic PTV cDNA clones: *in vitro* transcription, transfection, and phylogenetic analysis,” below. As no replication was detected following transfection of PTV-A11-(dC) RNA, rapid amplification of cDNA ends (RACE) was performed to determine missing 5′-terminal sequences of the viral genome.

**TABLE 1:** Oligonucleotides used in this study

Oligonucleotide	Sequence	Position (nt) in the complete genomic sequence
PTV-oligo(dC)_forw	5′-CCCCCCCCCCCCCCTCACCC-3′	120–139
Oligo(dT)-adapter	5′-GGCCACGCGTCGACTAGTACTTTTTTTTTTTTTTTTT-3′	7234–7252
Adapter	5′-GGCCACGCGTCGACTAGTAC-3′	–
(dT)	5′-TTTTTTTTTTTTTTTTT-3′	–
PTV-pBR-(dA)_forw	5′-AAAAAAAAAAAAAAAAAAAGTACTAGTCGACGCGTGGCCCCCG-3′	–
PTV-pBR-(dG)_rev	5′-GGGTGAGGGGGGGGGGGGGGCTATAGTGTCACCTAAATCGCG-3′	–
PTV-Dresden-5′-RACE-outer_rev	5′-CACCATTGGTGCAATACCTGGTG-3′	335–357
PTV-Gi2020-5′-RACE-outer_rev	5′-GTGCAATACCTGGTGACAATTGC-3′	327–349
PTV-Dresden-5′-RACE-inner_rev	5′-CACAACAAGAGACCGAGGTCTAG-3′	221–243
PTV-Gi2020-5′-RACE-inner_rev	5′-CAACAAGAGACCGAGGTCTAGTG-3′	219–241
PTV-3D_fwd	5′-TGGCATYCTCTGTTATGGTGATG-3′	6769–6791
PTV-5′-end_fwd	5′-TTCAAAGGGGGGGGGGTAGGGGCATC-3′	1–26
PTV-5′-SP6_rev	5′-CCCCCCCCTTGAAACTATAGTGTCACCTAAATCGCG-3′	1–15
Adapter-SpeI-pBR_fwd	5′-AGTCGACGCGTGGCCACTAGTCGACGCGTGGCCCCCGGGCAG-3′	–
Adapter-AclI-pBR_fwd	5′-AGTCGACGCGTGGCCAACGTTGTACTAGTCGACGCGGGCCCC-3′	–
Del-Adapter-AclI_fwd	5′-AAAAAAAAAAAAAAAACGTTGTACTAGTCGACGCGGGCCCC-3′	–
Del-Adapter-SpeI_fwd	5′-AAAAAAAAAAAAAAAGTACTAGTCGACGCGTGGCCCCCGGGCAG-3′	–
Del-Adapter_rev	5′-TTTTTTTTTTTTTTTTTTTTTTTTGCTCAACTA-3′	–
PTV-11-q_fwd	5′-CAGGATTGAGTGCGCTAATGTG-3′	705–726
PTV-11-q_rev	5′-GTTGTAGTTGATCACTCCAGT-3′	859–879
PTV-11_probe	5′-Fam-ATGTGGATCTTGAGATGCTACAGT-Tamra-3’	780–803
PTV-Gi2020-q_fwd	5′-CACCAGGTATTGCACCAATGGT-3′	336–357
PTV-Gi2020-q_rev	5′-CTGTCAGGCAGCACAAGTCCAG-3′	491–512
PTV-Gi2020_probe	Fam-5′-ACAGAAGAGCAAGTACTCCTGACT-3′-Tamra	369–392
mCherry_fwd	5′-GTGAGCAAGGGCGAGGAGGAT-3′	
mCherry_rev	5′-CTTGTACAGCTCGTCCATGCCG-3′	
mCherry-VP1_fwd	5′-ACCAGGATGTCATTTCAAGTGAGCAAGGGCGAGGAGGAT-3′	3368–3386
mCherry-PTV-A11-Nsps_fwd	5′-GGACGAGCTGTACAAGATGTCATTTCAAGGGCCGGGTGCC-3′	3373–3396
PTV-A11-Leader-mCherry_rev	5′-CTCGCCCTTGCTCACACCTTGGAACTGTAGCATCTCAAG-3′	788–810
PTV-11-VP1_rev	5′-TTGAAATGACATCCTGGTCATGTC-3′	3362–3385
Leader_deletion_fwd	5′-CAGTTCCAAGGTACTGGATCCTCC-3′	799–823
Leader_deletion_rev	5′-TCCAGTACCTTGGAACTGCATGGTGAGTTTCAACTGACTATA-3′	529–552
PTV-A11_13-16A_fwd	5′-GAAAATAGGGGCATCCGTTCGCGGATGT-3′	12–39
PTV-A11_13-16A_rev	5′-CTATTTTCCCCCCTTTGAACCTATAGTG-3′	1–19
PTV-A11_45-50A_fwd	5′-AAAAAATCTTTGGACCCAGCCATGGGTAAATG-3′	45–76
PTV-A11_45-50A_rev	5′-CCAAAGATTTTTTCAGAGACATCCGCGAACGG-3′	26–57

### RACE-PCRs

The 5′-terminal sequences of PTV-A11 (strain Dresden) and PTV-A Gi2020_1 were determined using 5′-RACE. Gene-specific primers were designed based on available UTR sequences. Total RNA was reverse transcribed using ProtoScript II Reverse Transcriptase (NEB) and gene-specific reverse primers (PTV-Dresden-5′-RACE-outer_rev or PTV-Gi2020-5′-RACE-outer_rev). The cDNA was purified and subjected to poly(A) tailing using terminal deoxynucleotidyl transferase (TdT; NEB). Tailed cDNA served as template for PCR amplification (OneTaq; NEB) using an Oligo(dT)-adapter primer in combination with the gene-specific reverse primers. Nested PCR was performed with adapter-specific and internal gene-specific primers (PTV-Dresden-5′-RACE-inner_rev or PTV-Gi2020-5′-RACE-inner_rev) yielding amplicons of 240 bps corresponding to an unknown 5′-terminal S-segment. PCR products were either directly sequenced or cloned into a pGEM-T vector (Promega, Madison, WI, USA) and sequenced using M13 primers. Due to difficulties in resolving homopolymeric sequences at the extreme 5′ terminus, an additional RNA circularization approach was employed. Total RNA was treated with thermolabile proteinase K (NEB) and converted to a 5′-monophosphate using RNA 5′pyrophosphohydrolase (RppH; NEB). Following purification, RNA was circularized using T4 RNA ligase 1 (NEB). Circular RNA served as a template for cDNA synthesis and semi-nested PCR using 5′-RACE primers in combination with a 3′-end-specific primer (PTV-3D_fwd). This approach enabled unambiguous determination of the 5′ terminus, revealing two uridine residues at the extreme 5′ end. The validated sequence was subsequently used for primer design in full-length genome amplification.

Secondary RNA structures of the S-segments of PTV-A11 (strain Dresden) and mengovirus (strain M; GenBank L22089.1) were predicted using the RNAfold web server (Vienna RNA websuite) (61). Secondary structures were calculated without constraints using both minimum free energy (MFE) prediction and partition function-based base-pairing probabilities, applying standard thermodynamic parameters for RNA (Turner model, 2004).

### Genomic PTV cDNA clones: *in vitro* transcription, transfection, and phylogenetic analysis

The validated 5′-end sequence was used for primer design in full-length genome amplification. Full-length cDNAs of PTV-A11 and PTV-A Gi2020_1 were generated by RT-PCR and cloned into a pBR322-derived vector backbone containing an SP6 promoter. For this purpose, viral RNA was reverse transcribed using an oligo(dT)-adapter primer. The resulting cDNA was purified and amplified by high-fidelity PCR (Q5; NEB) using gene-specific primers. Vector backbones were PCR-amplified to introduce overlapping sequences and restriction sites downstream of the poly(A) tail (SpeI for PTV-A11 and AclI for PTV-A Gi2020_1). Insert and vector fragments were assembled, and residual adapter sequences were removed by PCR-based deletion and homologous recombination. The final constructs were named PTV-A11-cDNA_pBR and PTV-A-Gi2020_1-cDNA_pBR. For *in vitro* transcription, plasmid DNA was linearized, purified, and transcribed. RNA transcripts were electroporated into SK-6 cells using a Gene Pulser II system (Bio-Rad, Hercules, CA, USA) at 0.18 kV and 950 µF. Cytopathic effects were observed within 72 h post transfection (hpt), and recombinant virus was harvested from the supernatant.

Nucleotide and amino acid sequences were aligned using CLC Genomics Workbench 20 (Qiagen) with default alignment parameters. Phylogenetic trees were constructed using the UPGMA method based on Jukes-Cantor distances. Tree robustness was assessed by bootstrap analysis with 1,000 replicates. Trees were visualized as unrooted phylograms.

### Subgenomic constructs, reporter viruses, and S-segment mutants

Subgenomic and mutant PTV-A11 cDNA constructs were generated by PCR-based deletion or insertion using PTV-A11-cDNA_pBR as template. Vector backbones were amplified using Q5 polymerase (NEB) with primers flanking the respective regions and introducing homologous overlaps for assembly. PCR products were recombined using DNA-assembly and resulting constructs were named according to the respective modification. For generation of a leaderless PTV construct, the complete leader coding sequence (252 bp; amino acids E_2_-F_85_) was deleted, yielding plasmid PTV-A11-ΔLeader_pBR. To ensure proper polyprotein processing, the resulting construct encoded an N-terminal MQFQ sequence upstream of VP4. To generate a replicative non-infectious subgenome, the structural protein coding region (2,562 bp; amino acids T_88_-R_941_) was replaced by an mCherry reporter gene, yielding PTV-A11-mCherry-ΔCPs_pBR. For the generation of infectious reporter viruses, a split mCherry system was employed. SK-6 cells stably expressing SpyCatcher fused to mCherry domains 1–10 were generated as described previously (62, 63). A corresponding viral construct encoding mCherry fragment 11 fused to a SpyTag sequence was inserted downstream of the 2A region (rPTV-A11-mCherry11-Spytag_pBR). A synthetic 2A sequence was included for proper processing (Insert sequence: P_947_YTIVEQYERAEGRHSTGGMDELYKATGSGSGSAHIVMVDAYKPTKNFSLLKQAGDVEEENPG). Infection of the reporter cells resulted in reconstitution of functional mCherry protein and fluorescence. In addition, a full-length fluorescent reporter virus was generated by insertion of the complete mCherry coding sequence between the P1 and P2 regions of the viral polyprotein, flanked by a duplicated 3C protease cleavage site. The resulting construct, PTV-A11-mCherry_pBR, produced replication-competent PTVs. To assess the functional relevance of RNA secondary structures within the S-segment, site-directed mutants were generated by substituting homopolymeric sequences (_13_GGGG_16_ to _13_AAAA_16_ and _45_CCCCCC_50_ to _45_AAAAAA_50_), yielding constructs PTV-A11-A_13-16__pBR and PTV-A11-A_45-50__pBR.

### Serum virus neutralization and interferon sensitivity assays

Serum virus neutralization assays (SVNAs) were performed as described previously (64). Serial fourfold dilutions of a PTV-A11-specific rabbit serum were prepared in triplicates in serum-free Dulbecco’s modified Eagle’s medium (DMEM) in 96-well plates. Virus stocks were adjusted to 500 TCID_50_ per 50 µL and incubated with the serum dilutions for 2 h at 37°C. PTV-A11 strain Dresden and rPTV-A11-mCherry were analyzed in parallel and back titrated on the same plate using fourfold serial dilutions. Subsequently, 1 × 10^4^ SK-6 cells were added per well, and plates were incubated for 72 h. Cells were fixed with 4% paraformaldehyde for 20 min at 4°C and stained with crystal violet. The mCherry fluorescence was quantified at 48 hpi using a Spark Cyto multimode reader (Tecan, Männedorf, Switzerland) and is presented as relative fluorescence units (RFU; excitation 575 nm, emission 621 nm). Neutralizing antibody titers were calculated as 50% neutralization doses (ND₅₀/mL) using the Spearman-Kärber method. For interferon (IFN) sensitivity assays, 1 × 10^6^ SK-6 cells were seeded in 6-well plates and pretreated with 500 IU human IFN-α2a (Roferon; Roche) for 24 h prior to infection or mock-treated. Cells were infected at a multiplicity of infection (MOI) of 0.01 for 1 h, followed by medium replacement including 500 IU human IFN-α2a. At 48 h post infection, cells were fixed and analyzed, and virus titers of supernatants were determined on naïve SK-6 cells without IFN-α2a.

### RT-qPCR analysis

RNA was extracted from whole-cell lysates or clarified supernatants using the QIAshredder and QIAamp Viral RNA Mini Kit on a QIAcube platform (Qiagen). Quantitative reverse transcription-PCR (RT-qPCR) was performed using the Luna Universal Probe One-Step RT-qPCR Kit (NEB) with strain-specific primer-probe sets (Table 1). Genome equivalents (GEs) were calculated using 7500 System SDS software (Applied Biosystems, Foster City, CA, USA) based on standard curves generated from *in vitro*-transcribed RNA. For viral growth analyses following transfection of synthetic PTV RNA, non-replicative subgenomes (PTV-A11-(dC)_pBR) were included as negative controls.

## RESULTS

### An incomplete 5′ terminus prevents recovery of infectious PTV-A11

Infection of SK-6 cells with the cell culture-adapted PTV-A11 strain Dresden resulted in rapid and widespread viral antigen expression in the monolayer (Fig. S1). In contrast, the field isolate PTV-A Gi2020_1 displayed delayed and limited infection at early time points. Despite these differences in kinetics, both viruses caused complete destruction of the cell monolayer within 72 h. To establish a reverse genetics system, we initially generated a full-length cDNA clone of PTV-A11 lacking sequences upstream of the poly(C) tract, analogous to a previously described PTV-A2 system (65). However, *in vitro*-transcribed RNA derived from this construct (PTV-A11-(dC)_pBR) failed to produce detectable viral protein expression following transfection (Fig. S2). In contrast, robust signals were observed in control infections. These findings indicate that the 5′ terminus of the PTV genome is incomplete in this construct and suggest the presence of essential upstream sequences. We, therefore, proceeded to characterize the 5′ end of the PTV genome in detail.

### Identification of the S-segment, secondary RNA structure, and phylogenetic analysis

**Fig 2:**
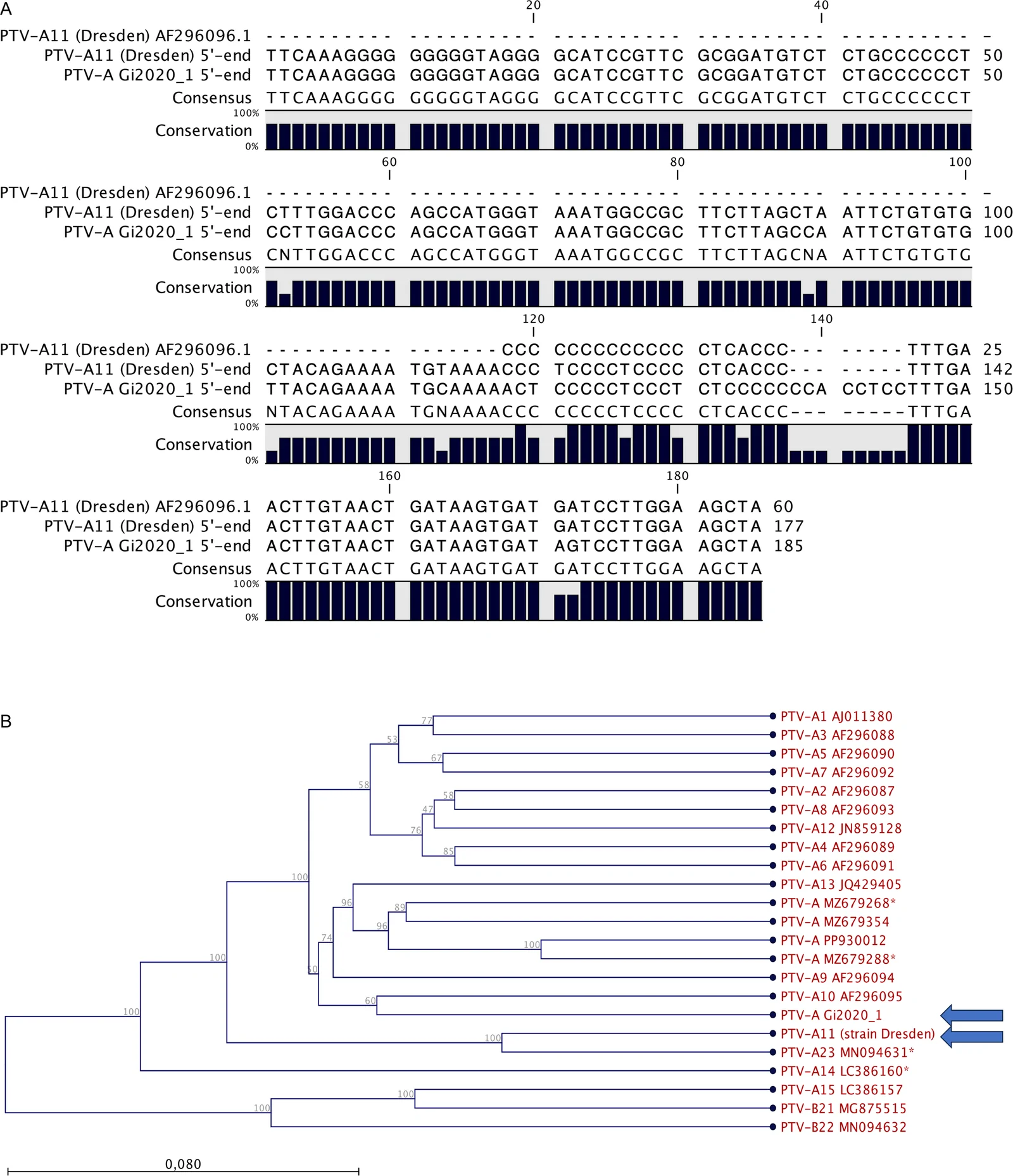
Sequence analyses of PTV-A11 (strain Dresden) and PTV-A Gi2020_1. (**A**) Alignment of the cDNA of S-segment, poly(C) tract, and the beginning of the L-segment of the 5′-UTR identified in this study with the corresponding UTR-segments of the PTV-A11 reference sequence (AF296096). The S-segments of PTV-A11 and PTV-A Gi2020_1 (117 or 118 nt) are nearly identical, differing at only four nucleotide positions, represented as “N” in the consensus sequence. Characteristic T and A insertions in the poly(C) region were identified, consistent with patterns described in other picornaviruses. (**B**) Phylogenetic analysis of PTV-A Gi2020_1 based on available complete or nearly complete genomes of representative strains of accepted serotypes of PTV-A and PTV-B, as well as newly proposed genotypes/serotypes and the closest relatives of PTV-A Gi2020_1 identified by BLAST search. The following sequences were included: PTV-A1, strain F65 (AJ011380); PTV-A2, strain T80 (AF296087); PTV-A3, strain O 2b (AF296088); PTV-A4, strain PS 36 (AF296089); PTV-A5, strain F 26 (AF296090); PTV-A6, strain PS 37 (AF296091); PTV-A7, strain F 43 (AF296092); PTV-A8, strain UKG/173/74 (AF296093); PTV-A9, strain Vir 2899/84 (AF296094); PTV-A10, strain Vir 460/88 (AF296095); PTV-A11, strain Dresden (AF296096 adapted plus S-segment); PTV-A12, strain CC25 (JN859128); PTV-A13, strain wild boar/WB2C-TV/2011/HUN (JQ429405); PTV-A14, strain JPN/MoI2-2-2/2015/G (LC386160); PTV-A15, strain JPN/Ishi-Ta2/2016/G (LC386157); PTV-A23, strain YC23 (MN094631); PTV-A, strain 276-k141_134203 (MZ679268); PTV-A, strain mt11vf_Pig (PP930012); PTV-A, strain 8-k141_255894 (MZ679354); PTV-A, strain 7-k141_304605 (MZ679288); PTV-B21, strain HuN41 (MG875515); PTV-B22, strain JiangX1 (MN094632). After sequence alignment, an unrooted decreasing phylogram was generated. Incomplete sequences are marked with an asterisk, and PTV-A Gi2020_1 as well as PTV-A11 are indicated by blue arrows. Branch labels show bootstrap values (%) from 1,000 replicates. A scale bar represents the number of substitutions per site.

5′-RACE analysis revealed a previously uncharacterized 5′-terminal sequence of 117–118 bp in both PTV-A11 and PTV-A Gi2020_1 (Fig. 2A). This sequence was highly conserved between the two strains and was independently confirmed by RNA circularization and cDNA tailing approaches. RNAfold analysis predicted the formation of a stable stem-loop structure within the 5′-proximal region of the PTV-A11 S-segment, whereas the adjacent poly(C) tract remained largely unstructured (Fig. S3). Although less pronounced than the well-characterized stem-loop of the mengovirus S-segment, the overall organization of the PTV-A11 S-segment is consistent with a conserved role of structured 5′-terminal RNA elements in picornavirus replication. Inclusion of the S-segment completes the PTV genome, resulting in total genome lengths of 7,231 nt for PTV-A11 and 7,241 nt for PTV-A Gi2020_1 excluding the poly(A) tail. Phylogenetic analysis based on full-genome sequences placed PTV-A Gi2020_1 outside established PTV-A genotypes (Fig. 2B). Because PTV-A Gi2020_1 showed only a distant relatedness to the established serotypes PTV-A10 (AF296095), PTV-A9 (AF296094), and PTV-A13 (JQ429405), we expanded the data set by adding the closest available matches identified via BLAST search (https://blast.ncbi.nlm.nih.gov/Blast.cgi?PROGRAM=blastn&PAGE_TYPE=BlastSearch&LINK_LOC=blasthome). However, even the most similar genomes (MZ679268, MZ679288, MZ679354, and PP930012) shared only about 84% nucleotide identity with our field isolate. This level of divergence is above the usual threshold between distinguished PTV serotypes. The closest related strains originated from East and Southeast Asia indicating substantial geographic and evolutionary separation (66). No evidence for recombination with known PTV strains was detected. Together, the observed genetic divergence and distinct phylogenetic clustering suggest that PTV-A Gi2020_1 represents a previously unrecognized genotype, potentially corresponding to a novel serotype pending serological confirmation. In accordance with ICTV guidelines, cross-neutralization assays would be required to formally determine the serotype classification, but this is beyond the scope of this work.

### Completion of the 5′-end enables recovery of infectious PTV clones

**Fig 3:**
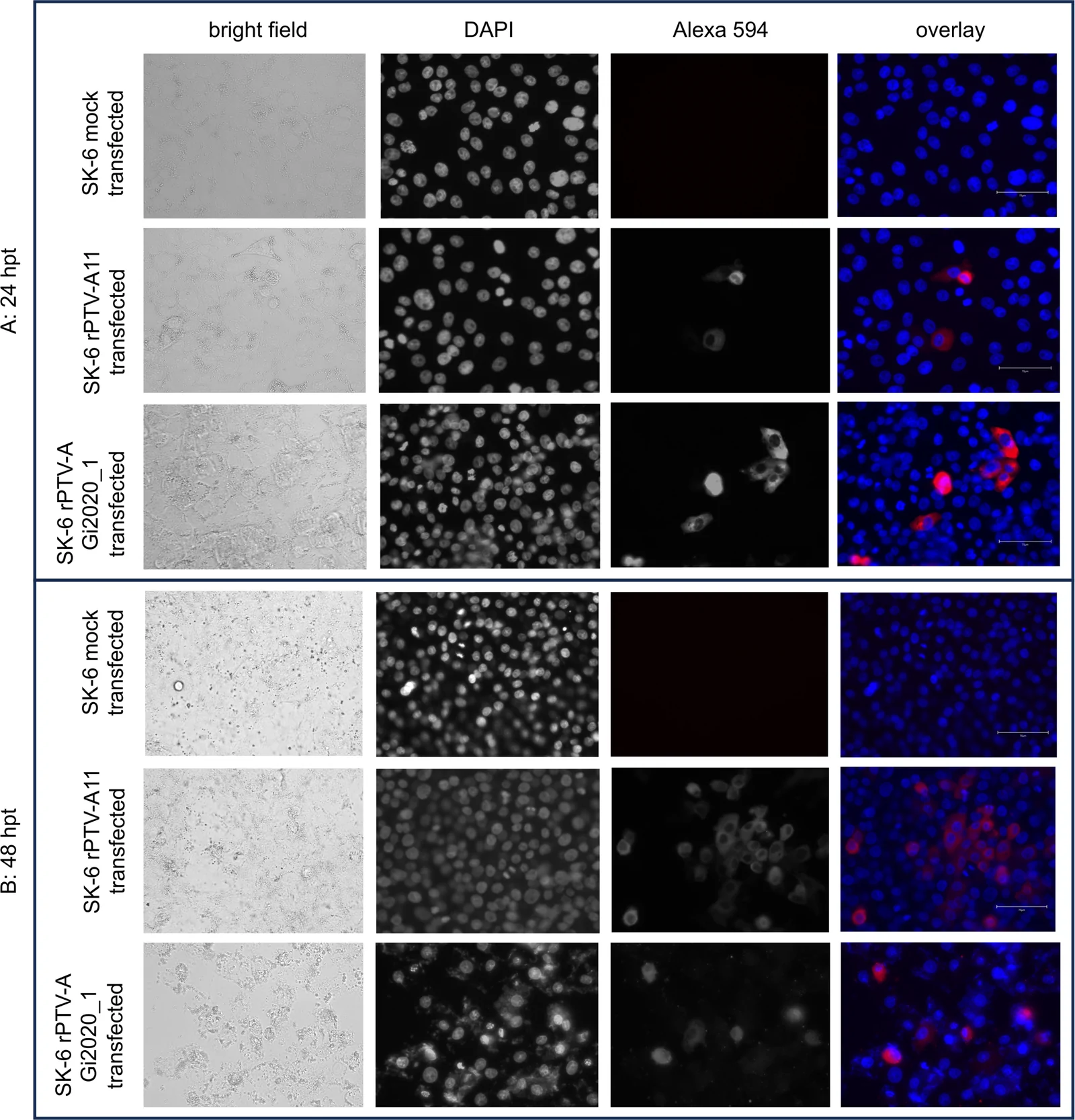
Recovery of recombinant PTVs from infectious cDNA clones using *in vitro*-transcribed RNAs. (**A**) At 24 hpt, viral capsid protein expression was detected in individual transfected cells, displaying the characteristic cytoplasmic staining pattern of PTV infection. (**B**) At 48 hpt, both recombinant viruses had spread to neighboring cells and formed distinct infection foci. Cells were stained with the PTV-specific monoclonal antibody 4B1 and an Alexa Fluor 594-conjugated goat anti-mouse secondary antibody. Nuclei were counterstained with DAPI. Representative overlay images are shown including virtual scale bars (75 µm). All images were taken at 40× magnification.

**Fig 4:**
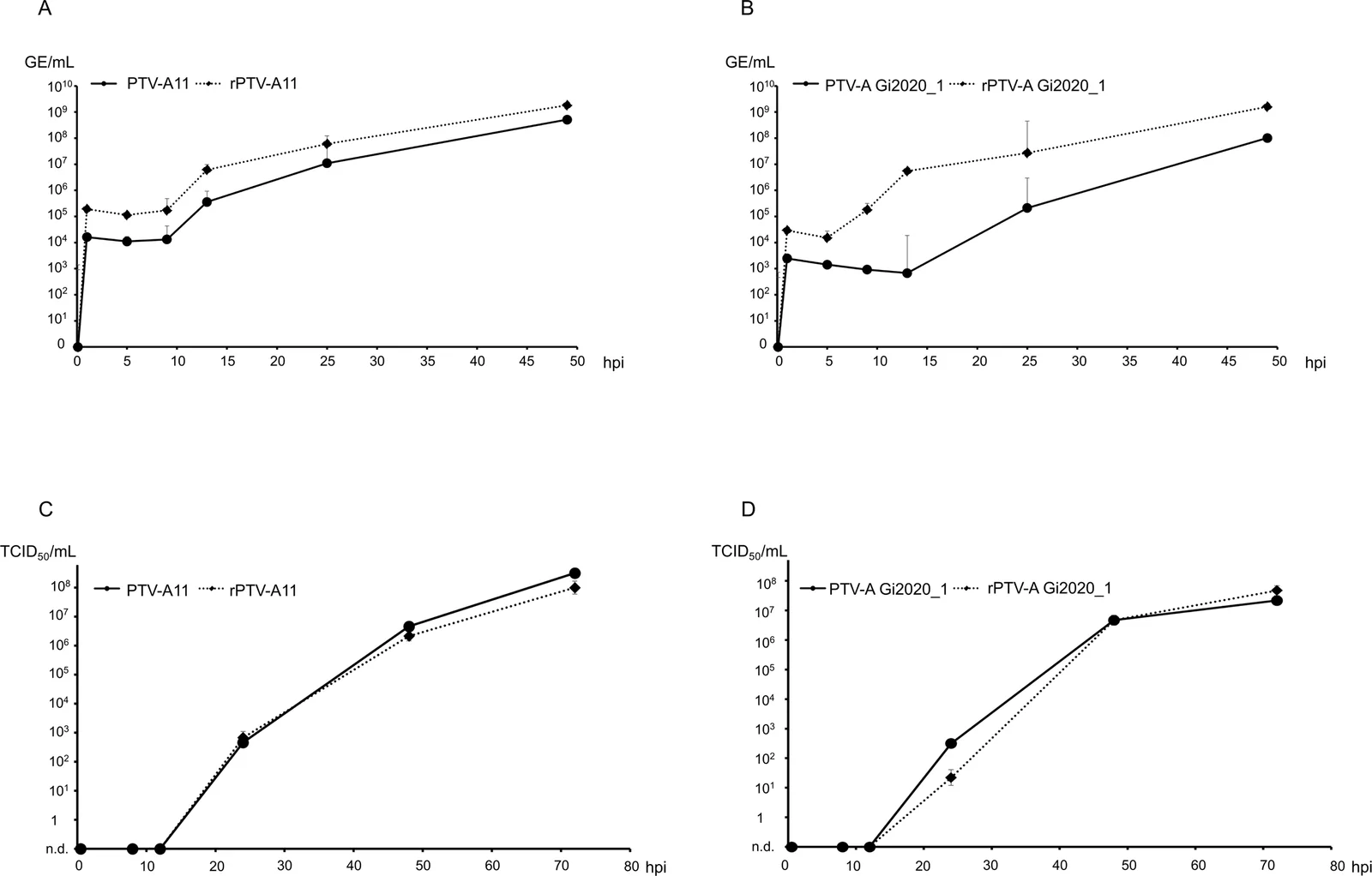
Intracellular RNA replication and growth kinetics of parental and cDNA-derived PTVs. (**A and B**) Intracellular RNA replication kinetics of parental virus stocks and the corresponding cDNA-derived viruses rPTV-A11 (**A**) and rPTV-A Gi2020_1 (**B**). SK-6 cells were infected at a MOI of 1.0. After 1 h of adsorption, the inoculum was removed, unattached virions were washed away with PBS, and fresh medium was added. At the indicated time points, cells were harvested and lysed for RNA extraction. Viral RNA levels were quantified by RT-qPCR assays and expressed as genome equivalents per ml of cell lysate. RNA replication kinetics were highly similar between parental and recombinant viruses although the cDNA-derived viruses consistently exhibited approximately 1 log₁₀ higher RNA levels. (**C and D**) Multi-step growth kinetics of parental virus stocks and the corresponding cDNA-derived virus clones rPTV-A11 (**C**) and rPTV-A Gi2020_1 (**D**). SK-6 cells were infected at an MOI of 0.01. After 1 h of adsorption, the inoculum was removed, unattached virions were washed away with PBS, and fresh medium was added. At the indicated time points, culture supernatants were collected and virus titers were determined by TCID_50_ assay. Recombinant virus clones displayed growth kinetics comparable to those of their parental counterparts. The growth curves shown are representative of a single comparative virus growth experiment in which parental and cDNA-derived viruses were analyzed in parallel under identical experimental conditions. Virus titers were determined by TCID_50_ assays performed in technical triplicates. Statistical comparisons of progeny virus titers were performed separately using data obtained from three independent biological experiments.

Full-length cDNA clones of PTV-A11 and PTV-A Gi2020_1 were generated from single RT-PCR amplicons and sequenced including the newly identified 5′-terminal S-segment (Fig. S4 and S5). The sequence of the PTV-A11 clone (rPTV-A11) was nearly identical to the published reference sequence, differing only by a small number of nucleotide substitutions and a serine insertion. Despite substantial sequence divergence at the nucleotide level (only about 82% identity), the PTV-A Gi2020_1 clone (rPTV-A Gi2020_1) retained high amino acid similarity with rPTV-A11 and an overall conserved polyprotein organization, consistent with its classification as a PTV-A strain. Compared to rPTV-A11, it presents an identical polyprotein length with only one sequence gap of a single amino acid each (Q insertion behind D_338_ and D_933_ deletion in PTV-A Gi2020_1; numbering referring to PTV-A11). *In vitro*-transcribed RNAs derived from both full-length clones produced robust viral protein expression following transfection of SK-6 cells. Infected cells were detectable by immunofluorescence at 24 hpt, followed by plaque formation within 48 h (Fig. 3). These results demonstrate that inclusion of the complete 5′-terminus restores infectivity and enables recovery of recombinant PTV from cloned cDNA. RNA transfection of both PTV clones resulted in low initial transfection efficiencies, with viral protein expression detected in approximately 1% of cells at 24 hpt. Despite this, both viruses spread rapidly and established productive infections. Comparative analyses revealed similar replication kinetics between cDNA-derived viruses and their parental counterparts, as determined by intracellular RNA quantification and multistep growth curves (Fig. 4). Both recombinant viruses reached titers exceeding 10^7^ TCID_50_/mL and displayed growth characteristics comparable to the respective parental strains. Although direct quantitative comparison between the polyclonal parental virus stock PTV-A11 strain Dresden and the clonal rPTV-A11 derived from the infectious cDNA is of limited biological relevance, progeny virus titers of rPTV-A11 (mean 9.3 × 10^7^ TCID_50_/mL, SD 5.8 × 10^7^) were not significantly different (*P* = 0.0665) from those of the parental PTV-A11 stock (mean 3.7 × 10^8^ TCID_50_/mL, SD 8.5 × 10^7^).

To assess the functional importance of the predicted stem-loop structure within the S-segment, recombinant PTV genomes carrying adenosine substitutions in homopolymeric regions expected to disrupt base pairing were generated (G_13–16_A and C_45–50_A, see Fig. 1B). RNAfold analysis predicted that the rPTV-A11-A_13–16_ mutant retained the overall stem-loop architecture despite a less favorable folding energy (MFE of −50.20 kcal/mol). Consistent with this prediction, the mutant remained replication competent but exhibited markedly delayed growth, with first antigen-positive cells detected at 24 hpt, but no visible virus spread at 48 hpi (Fig. S6). In contrast, the C_45–50_A substitutions abolished the predicted stem-loop architecture of PTV-A11 and resulted in an alternative RNA fold comprising multiple short hairpins in a circular arrangement. Despite an overall more favorable folding energy (MFE of −59.50 kcal/mol), rPTV-A11-A_45–50_ was non-replicative, as no viral antigen could be detected following RNA transfection or subsequent passage of transfected cells. These findings indicate that the integrity of the 5′-proximal stem-loop structure, rather than overall RNA folding energy or nucleotide sequence, is critical for PTV replication and support a functional role of the PTV S-segment as a cis-acting RNA element.

### Purification of PTV-A Gi2020_1 virions and analysis of capsid protein processing

**Fig 5:**
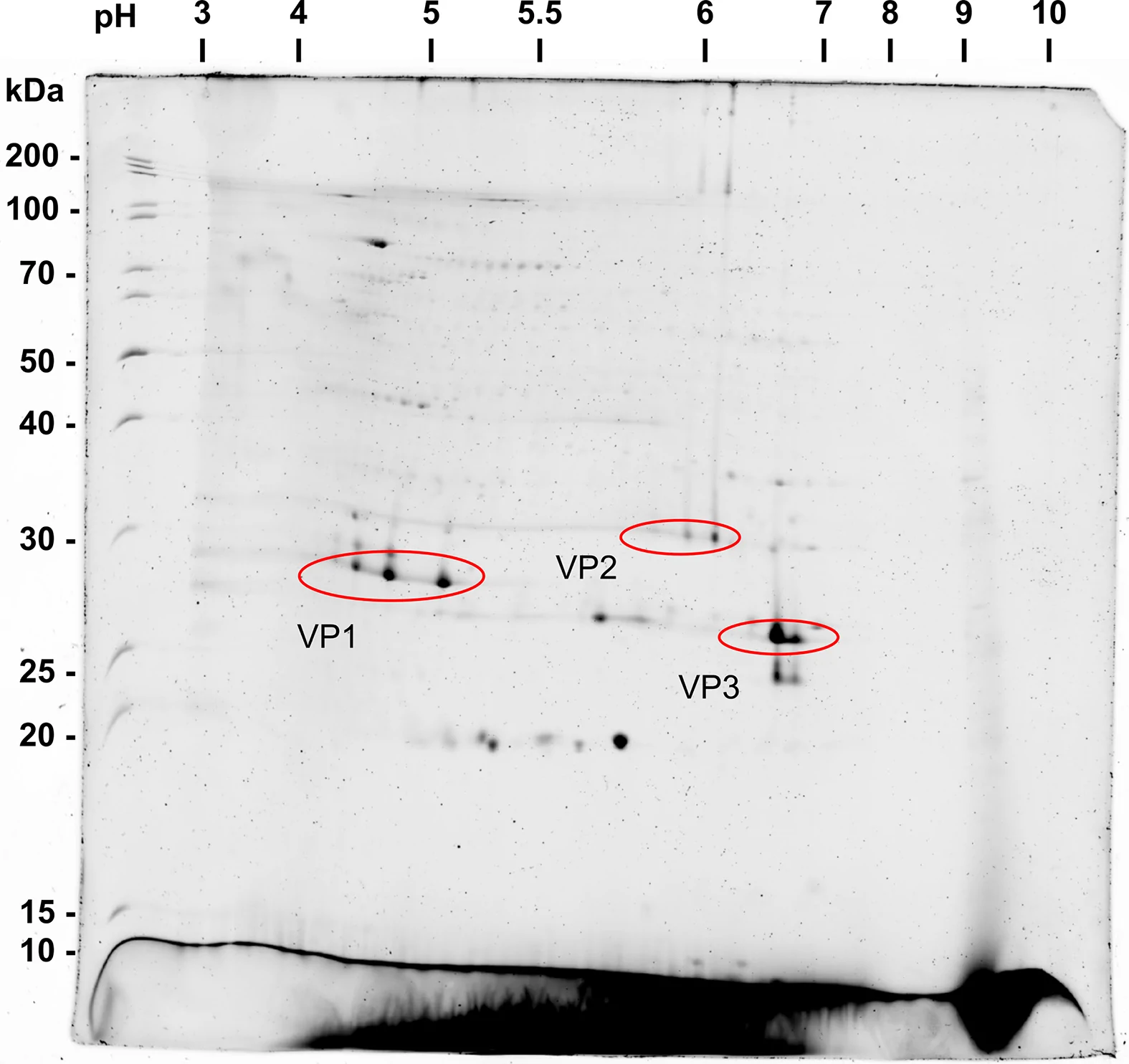
Two-dimensional SDS-PAGE and identification of PTV-A Gi2020_1 capsid proteins. Viral proteins were separated by isoelectric focusing (pH 3–10) followed by SDS-PAGE and transferred to a PVDF membrane. Prominent Flamingo-stained protein spots (shown in grayscale for improved contrast) were excised and subjected to MALDI-TOF mass spectrometry. Spots identified as VP1, VP2, and VP3 are indicated. Additional protein spots detected in the preparation were not assigned to viral structural proteins. The horizontal axis indicates the isoelectric point (pI), and the vertical axis indicates the molecular mass (kDa).

PTV virions were enriched from infected cell culture supernatants by ultracentrifugation and CsCl gradient separation. Infectious particles and viral antigen were predominantly recovered in fractions with buoyant densities of 1.30–1.35 g/mL, consistent with known picornavirus properties (Fig. S7) (67). To facilitate preparative purification, a step-gradient approach was established, yielding highly concentrated virion preparations suitable for downstream analyses. Capsid proteins of PTV-A Gi2020_1 were analyzed by 2D SDS-PAGE and mass spectrometry (Fig. 5). The calculations and numerical values presented here refer to the nucleic acid sequence and the deduced amino acid sequence of PTV-A Gi2020_1 (MW824925). VP2 (E_161_-G_441_, calculated molecular mass: 31.3 kDa, pKi: 6.70) was identified in the 2D SDS-PAGE as three spots of 32 kDa with pKis between 5.7 and 6.1. Peptide mass fingerprinting of the VP2 spots yielded a sequence coverage of 55.87% although the extreme N- and C-terminal regions were not represented. The abundance of lysine and arginine residues near both termini likely resulted in the generation of very short tryptic peptides that were not detected by MALDI-TOF-MS. VP3 (G_442_-Q_682_, calculated molecular mass: 26.8 kDa, pKi: 7.70) was identified in the 2D SDS-PAGE as two spots of 27 kDa with pKis of 6.5 and 6.6. The peptide mass fingerprints of the VP3 spots yielded in a protein coverage of 89.67%. The very small N-terminal peptide (GPVPK) was not detected, but the C-terminal peptide was found uncovering the polyprotein processing site (Q_683_/G). VP1 (G_684_-Q_945_, calculated molecular mass: 28.5 kDa, pKi: 5.47) was identified in the 2D SDS-PAGE as three spots of a 28 kDa protein with pKis between 4.5 and 5.2. The peptide mass fingerprints of the VP1 spots covered 68.70% of the protein sequence including N- and C-terminus. This confirmed the G_684_/Q cleavage site at the N-terminus and revealed the C-terminal cleavage site Q_945_/G. The small VP4 (G_87_-A_160_, calculated molecular mass: 7.3 kDa, pKi: 4.77) was not detected in the 2D SDS-PAGE, as proteins of this size are not resolved under the SDS-PAGE conditions used. Peptides of VP4 were also not represented in direct peptide mass fingerprinting analyses of our virus preparations, whereas VP1, VP2, and VP3 were consistently identified. However, this is also consistent with the small size and basic nature of VP4, which result in few informative peptides upon tryptic digestion, precluding reliable detection by mass spectrometry. Since the identified N- and C-terminal peptides of the PTV structural proteins could not have been generated by trypsin cleavage, our data confirmed parts of the proposed cleavage pattern of the 3C-protease in the PTV-A Gi2020_1 polyprotein. The data support a cleavage pattern involving sites at L/VP4 (Q_86_/G), VP4/VP2 (A_160_/E), VP2/VP3 (E_441_/G), VP3/VP1 (Q_683_/G), and VP1/2A (Q_945_/G). Together, the data provide experimental support for a conserved polyprotein processing scheme of PTV-A.

### Functional analysis of the leader protein of PTV-A11

**Fig 6:**
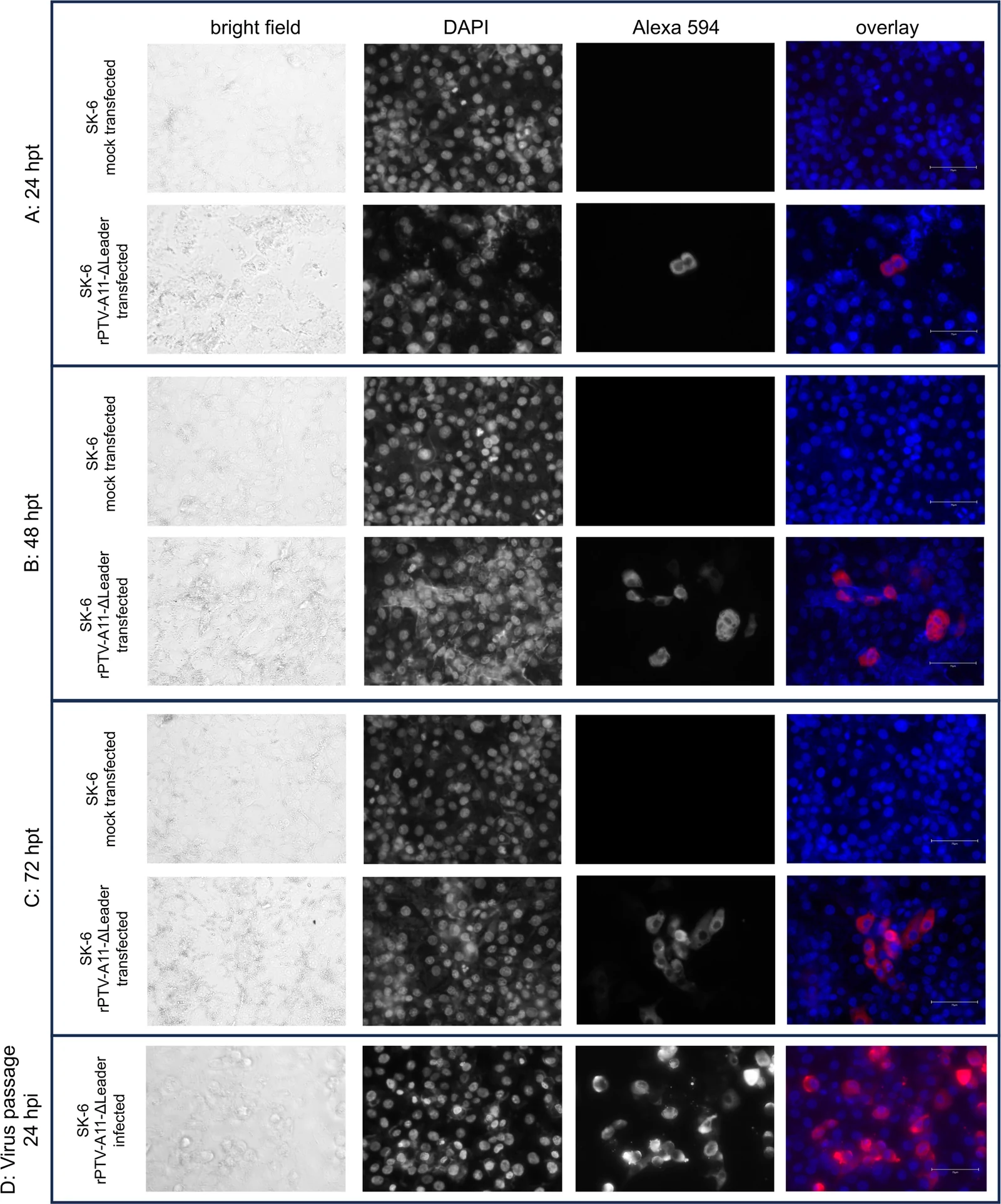
Replication and spread of the leaderless virus rPTV-A11-ΔLeader. Indirect immunofluorescence analysis of SK-6 cells transfected with *in vitro*-transcribed rPTV-A11-ΔLeader RNA. (**A**) At 24 hpt, viral capsid protein expression was detected in individual transfected cells, displaying the characteristic cytoplasmic staining pattern of PTV infection. (**B**) At 48 hpt, rPTV-A11-ΔLeader had spread to neighboring cells. (**C**) At 72 hpt, distinct infection foci and cytopathic effects were observed. (**D**) Representative immunofluorescence image of rPTV-A11-ΔLeader after serial passaging, demonstrating continued production of infectious progeny virus, even though rPTV-A11-ΔLeader showed significantly slower growth compared to the wild-type genome. Cells were stained with the PTV-specific monoclonal antibody 4B1 and an Alexa Fluor 594-conjugated goat anti-mouse secondary antibody. Nuclei were counterstained with DAPI. Representative overlay images are shown. Scale bar, 75 µm with original magnification of 40×.

To investigate the role of the PTV Leader protein, a leaderless PTV-A11 mutant (rPTV-A11-ΔLeader) was generated. Following RNA transfection, viral protein expression and plaque formation were observed, indicating that the Leader is dispensable for genome replication and morphogenesis in cell culture (Fig. 6). Despite retaining the ability to replicate and spread in cell culture, rPTV-A11-ΔLeader exhibited markedly reduced growth reaching titers of approximately 10^5^ TCID_50_/mL (Fig. 9C). Statistical analysis of progeny virus titers obtained from three independent biological experiments confirmed that attenuation of rPTV-A11-ΔLeader (mean 1.3 × 10^5^ TCID_50_/mL, SD 4.5 × 10^4^) was highly significant compared with rPTV-A11 (*P* < 0.0001, Fig. 9D). This attenuated phenotype remained stable over serial passaging, with no evidence of reversion as confirmed by full-genome sequencing after 10 passages. Pretreatment of SK-6 cells with IFN-α2a for 24 h effectively abrogated infection by rPTV-A11-ΔLeader, with only a single antigen-positive cell detected (Fig. S8). In contrast, IFN-α2a pretreated cells remained partially permissive to the wild-type PTV-A11 although viral spread and plaque formation were clearly suppressed. These results suggest that the PTV leader protein is a virulence factor that promotes the efficient production of progeny viruses by counteracting the innate immune response, while it is not essential for genome replication or morphogenesis in cultured cells.

### Generation and characterization of a replication-competent PTV-A11 replicon

**Fig 7:**
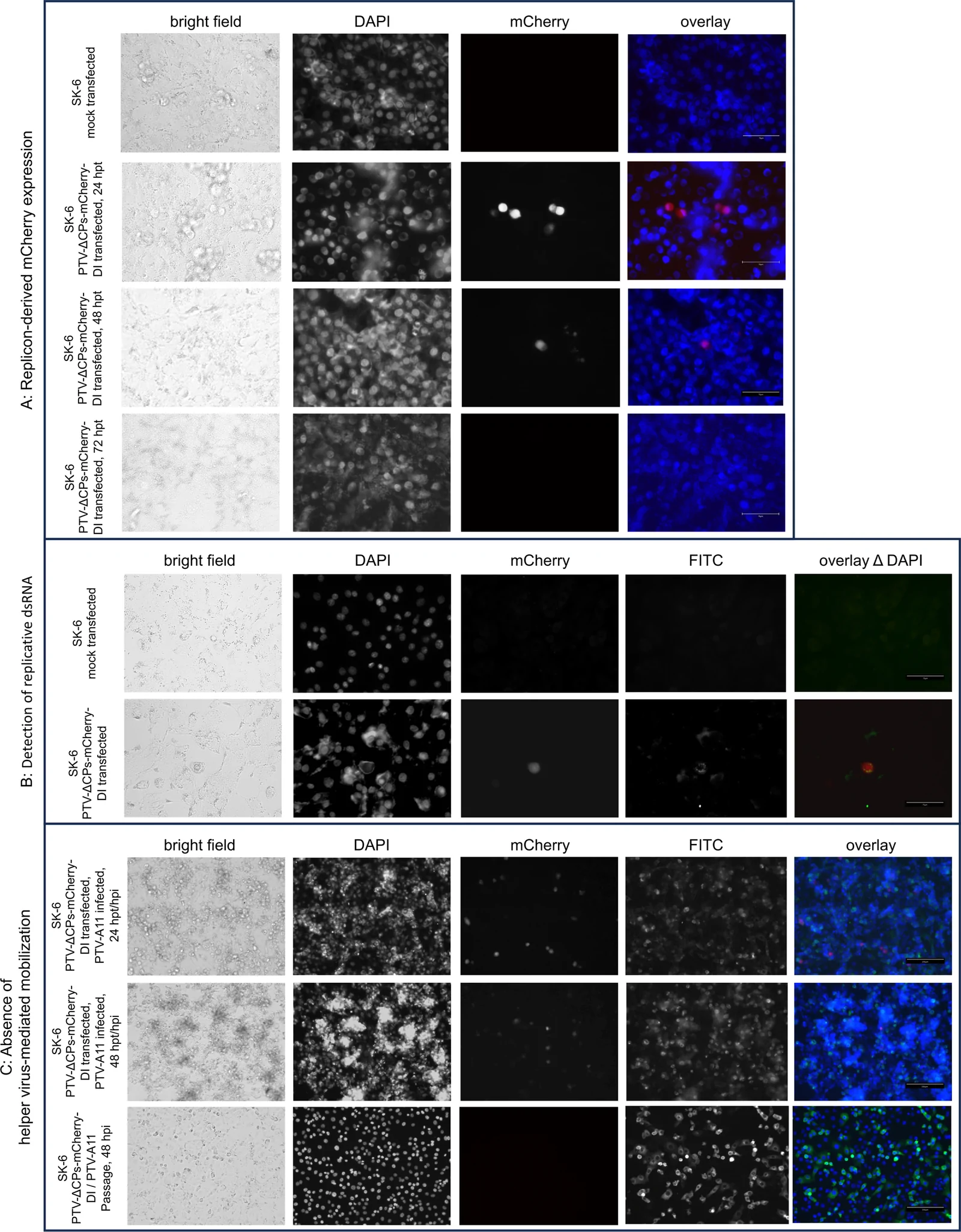
Reporter expression, dsRNA replication intermediates, and lack of helper virus-mediated mobilization of the replicon rPTV-A11-mCherry-ΔCPs. (**A**) Expression of the mCherry reporter following transfection of rPTV-A11-mCherry-ΔCPs RNA. At 24 hpt, strong cytoplasmic and nuclear fluorescence signals characteristic of freely diffusible mCherry were detected in transfected cells. Reporter fluorescence declined at later time points owing to the cytopathic effects associated with replicon replication. (**B**) Detection of replicative double-stranded RNA (dsRNA) intermediates in rPTV-A11-mCherry-ΔCPs-transfected cells at 24 hpt. The dsRNA was visualized using monoclonal antibody J2 and appeared as punctate cytoplasmic signals in cells expressing the mCherry reporter. A weak diffuse background signal was occasionally observed in mCherry-negative cells following J2 staining but was clearly distinguishable from the punctate cytoplasmic dsRNA signal associated with replicon replication. (**C**) Analysis of mobilization of rPTV-A11-mCherry-ΔCPs genomes by a helper virus. SK-6 cells transfected with the replicon were superinfected with PTV-A11 at a MOI of 1.0. No mCherry-positive infection foci were detected at 48 hpt/hpi. No reporter fluorescence was observed in naïve cells inoculated with supernatants from replicon-transfected and helper virus-infected cultures indicating the absence of detectable trans-packaging. Nuclei were counterstained with DAPI, and representative overlay images are shown. For dsRNA detection, DAPI staining was omitted from the overlay to improve visualization of the J2 signal. Scale bars, 75 µm or 150 µm are indicated with original magnifications of 20× and 40×.

To dissect PTV RNA replication from progeny virus production, a subgenomic replicon was generated by replacing the structural protein coding region with an mCherry reporter (rPTV-A11-mCherry-ΔCPs). This construct supported RNA replication, as indicated by reporter expression in transfected cells and detection of double-stranded RNA (Fig. 7), but did not produce infectious virus particles. Replicon-containing cells exhibited cytopathic effects and were not maintained beyond 72 hpt. Superinfection with wild-type PTV-A11 did not result in replicon mobilization, as no plaques with reporter expression were detected following transfection and infection or during subsequent passages. These findings suggest that genome encapsidation is tightly regulated and may occur in spatially restricted subcellular compartments. Together, we established a replication-competent subgenomic system of PTV-A11 for functional studies of PTV replication.

### Infectious reporter viruses of PTV-A11

To establish fluorescent reporter systems for an infectious PTV, two independent strategies were evaluated. First, a split-mCherry system was implemented by inserting a minimal reporter cassette encoding mCherry domain 11 fused to a SpyTag downstream of the 2A region (rPTV-A11-mCherry11-SpyTag, see Fig. 1B). In combination with a reporter cell line expressing the complementary mCherry1-10-SpyCatcher fragment, this system enabled reconstitution of fluorescence upon infection. Although reporter activity was detectable and the viral genome remained genetically stable over serial passaging, replication was severely impaired, resulting in very low titers of about 10^2^ TCID_50_/mL and limited spread (Fig. S9). Even the serial passaging of transfected cells did not improve the replication efficiency of the construct.

**Fig 8:**
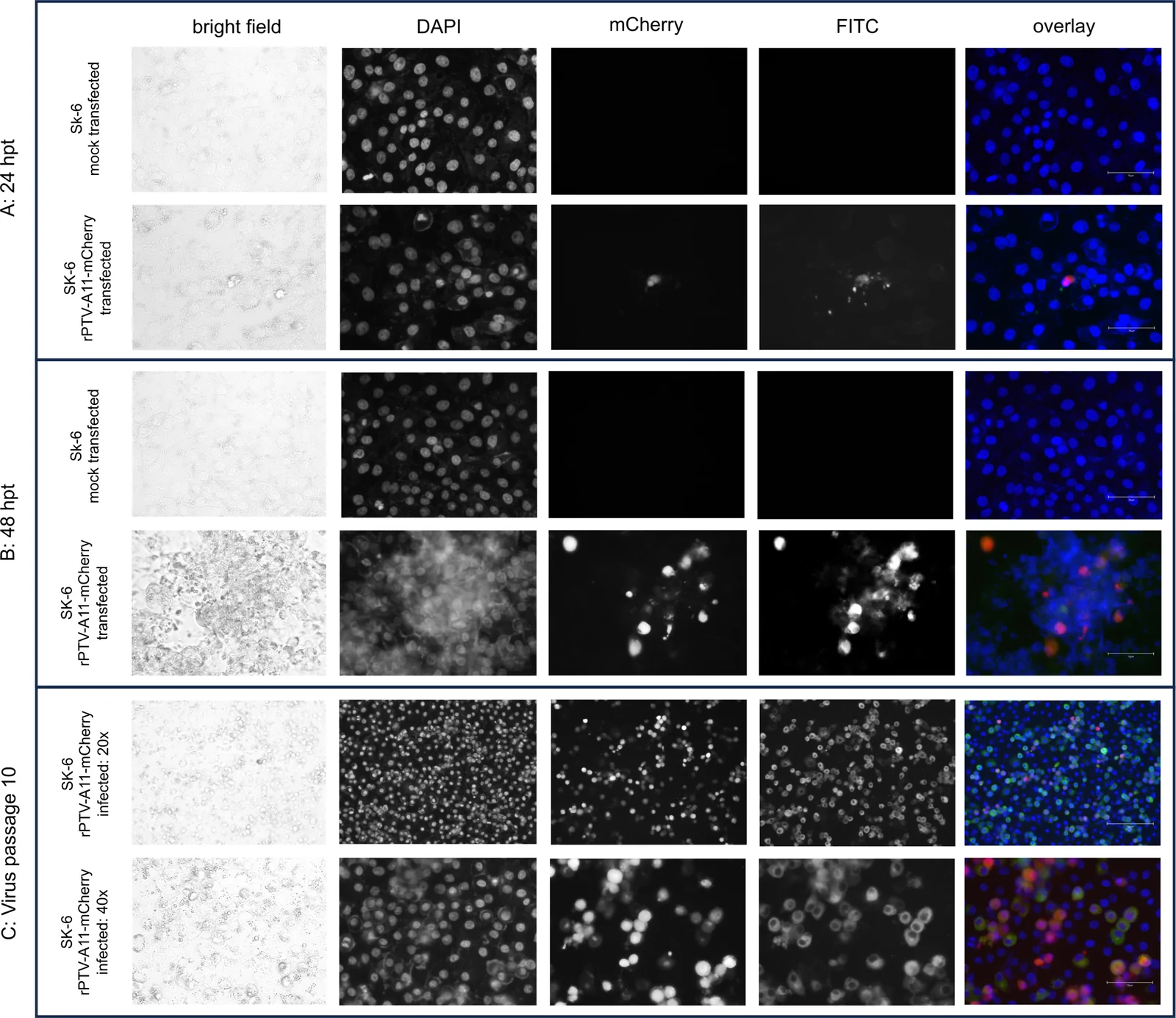
Characterization of the infectious reporter virus rPTV-A11-mCherry. (**A**) At 24 hpt, mCherry fluorescence and PTV antigen expression were detected in individual cells transfected with rPTV-A11-mCherry RNA. (**B**) At 48 hpt, both mCherry fluorescence and immunofluorescence signals demonstrated spread of the reporter virus to neighboring cells. (**C**) Genetic stability of rPTV-A11-mCherry after 10 serial passages. Reporter fluorescence remained detectable in all infected cells, indicating stable maintenance of the mCherry reporter gene. Nuclei were counterstained with DAPI. Representative overlay images are shown. Scale bars, 75 µm or 150 µm as indicated with original magnifications of 20× and 40×.

**Fig 9:**
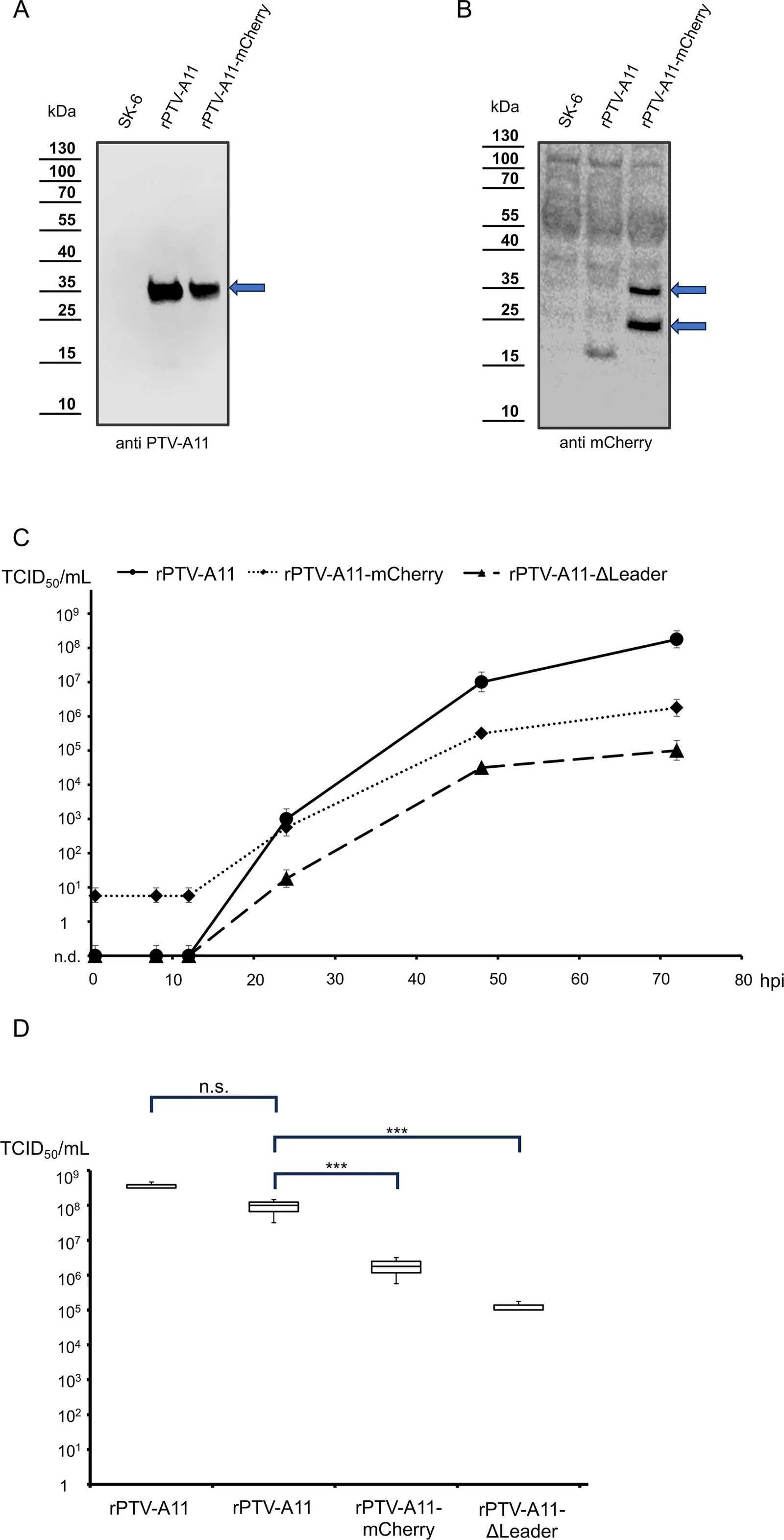
Protein expression and growth characteristics of recombinant PTV-A11 viruses. (**A**) Western blot analysis of rPTV-A11 and rPTV-A11-mCherry infected cells using a PTV-A11-specific rabbit antiserum. The VP1 signal is indicated by an arrow. (**B**) Western blot detection of mCherry expression in rPTV-A11-mCherry infected cells using an mCherry-specific monoclonal antibody. Signals were detected at approximately 25 and 35 kDa (arrows). The higher-molecular-weight species is consistent with an incompletely processed mCherry-2A/2B fusion protein. (**C**) Multi-step growth kinetics of rPTV-A11, rPTV-A11-mCherry, and rPTV-A11-ΔLeader following infection of SK-6 cells at a MOI of 0.01. Both mutant viruses exhibited growth kinetics comparable to rPTV-A11 but reached peak titers approximately 2–3 log₁₀ lower than the parental virus. The growth curves shown represent a single comparative virus growth experiment in which all viruses were analyzed in parallel under identical experimental conditions. Virus titers were determined at the indicated time points by TCID_50_ assays performed in technical triplicates. (**D**) Statistical analysis of progeny virus titers at 72 hpi from three independent biological experiments demonstrated that both rPTV-A11-mCherry and rPTV-A11-ΔLeader produced significantly lower titers than rPTV-A11. Statistical significance was determined by one-way ANOVA followed by Tukey’s multiple-comparison test (n.s., not significant; ***, *P* < 0.001).

In contrast, insertion of a full-length mCherry gene between the structural and non-structural regions, flanked by duplicated 3C protease cleavage sites, yielded a robust infectious reporter virus (rPTV-A11-mCherry, see Fig. 1B). This construct displayed strong fluorescence, efficient cell-to-cell spread, and pronounced cytopathic effects (Fig. 8). Although rPTV-A11-mCherry reproducibly reached high titers (mean 1.8 × 10^6^ TCID_50_/mL, SD 1.3 × 10^6^), progeny virus production was significantly lower than those of rPTV-A11 (*P* = 0.0003, Fig. 9C and D). The genome remained stable over at least 10 passages, with no alterations detected in the reporter gene by full-genome re-sequencing. These results demonstrate that PTV tolerates insertion of a full-length fluorescent protein at the P1-P2 junction, whereas even small insertions at alternative sites can severely compromise viral fitness. The rPTV-A11-mCherry virus, therefore, represents a robust platform for functional assays, including antiviral testing and neutralization studies. Consistent with this, side-by-side neutralization assays using a PTV-A11-specific rabbit antiserum yielded comparable results for wild-type PTV-A11 and rPTV-A11-mCherry at an equivalent virus dose. However, infection with the reporter virus can be quantified directly via fluorescence, enabling rapid and objective readouts without the need for immune- or RT-PCR detection (Fig. S10).

## DISCUSSION

The global pig industry is highly vulnerable to epidemic outbreaks of emerging pathogens due to the industrial-scale nature of piglet production and fattening, the regional clustering of large pig farms, and extensive international trade networks. Recent decades have seen frequent outbreaks of emerging diseases, such as African and classical swine fever, porcine epidemic diarrhea, and the porcine reproductive and respiratory syndrome underscoring an ongoing risk of viruses spreading rapidly through pig populations (68). PTVs are globally distributed pathogens of swine that typically cause mild endemic infections but retain the capacity to induce severe neurological disease. Although highly virulent strains such as the historical Teschen virus have become rare, the continuous circulation of genetically diverse PTVs and the potential for emergence of neuroinvasive variants remain a concern for porcine health and livestock production (69, 70).

A central finding of this study is the identification of a previously uncharacterized 5′-terminal S-segment in the PTV genome. This element, comprising 117–118 nucleotides, is highly conserved between two distinct PTV genotypes and proved essential for viral replication. Despite the early identification of teschoviruses back in the 1930s, the 5′-end of their genome remained understudied and reverse genetics were not established although the polyprotein-coding regions of PTVs are well characterized (7). A single study by Yuying Li et al. presented a reverse genetics system for a PTV-A2 (strain GX/2020, OM281048) reporting a genome structure beginning with a poly(C) tract and lacking the S-segment identified here (65). Our findings clearly demonstrate that PTV-A11 and PTV-A Gi2020_1 harbor a typical S-segment in their 5′-UTR, resolving a long-standing gap in teschovirus genome organization including earlier ambiguities. Beyond its biological relevance, this conservation may provide a useful target for future molecular diagnostics. We also identified the first functional poly(C) tract sequences of teschoviruses with 21 nts for PTV-A11 (C_118_CCUCCCCUCCCCCUCACCC_137_) and 27 nts for PTV-A Gi2020_1 (5′-C_119_UCCCCCUCCCUCUCCCCCCCACCUCC_145_-3’). Both poly(C) tracts are rather short and include uridine residues in characteristic alternations. It remains a question for future studies whether these short poly(C) tract sequences were selected by RT-PCR and during molecular cloning and what role the poly(C) tract plays in the virulence of teschoviruses.

**Fig 10:**
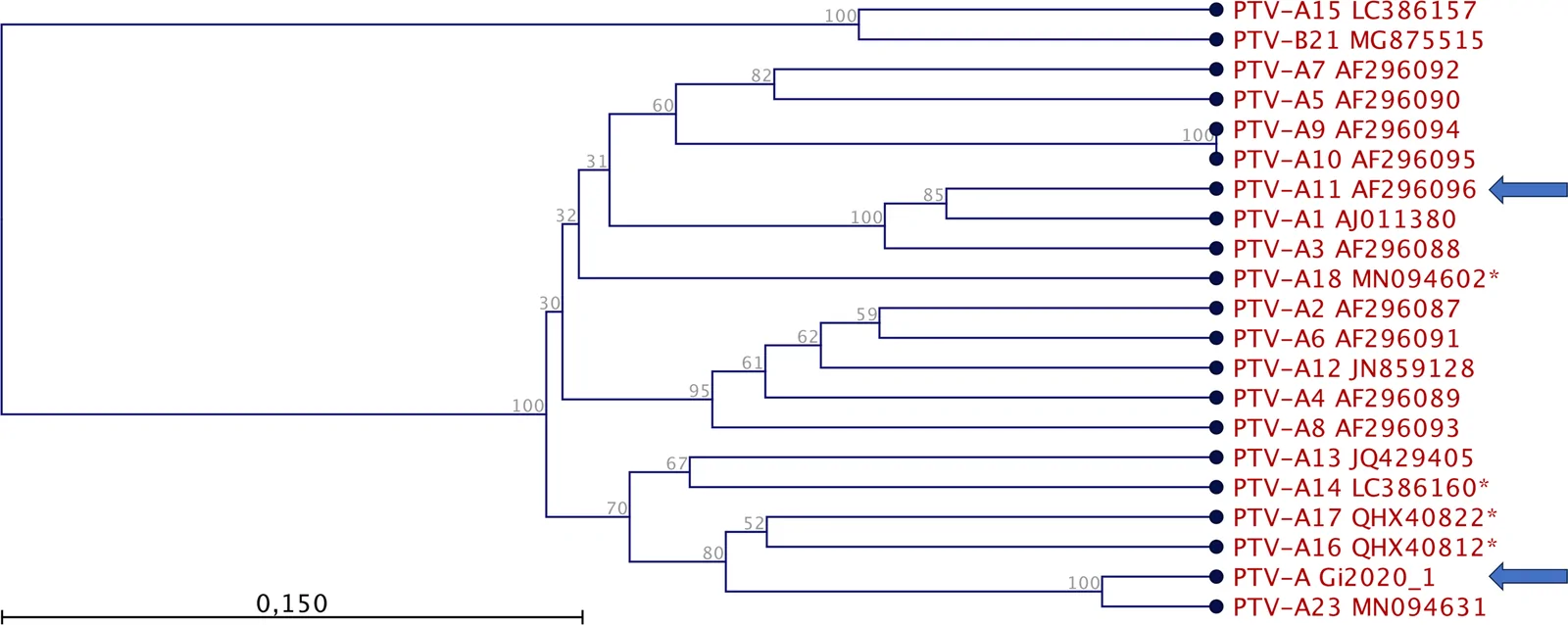
Phylogenetic analysis of the VP1 protein sequence of PTV-A Gi2020_1. Amino acid sequences of VP1 from representative strains of accepted PTV-A and PTV-B serotypes, together with recently proposed genotypes/serotypes, were aligned and used to generate an unrooted phylogram. The following sequences were included: PTV-A1, strain F65 (AJ011380); PTV-A2, strain T80 (AF296087); PTV-A3, strain O 2b (AF296088); PTV-A4, strain PS 36 (AF296089); PTV-A5, strain F 26 (AF296090); PTV-A6, strain PS 37 (AF296091); PTV-A7, strain F 43 (AF296092); PTV-A8, strain UKG/173/74 (AF296093); PTV-A9, strain Vir 2899/84 (AF296094); PTV-A10, strain Vir 460/88 (AF296095); PTV-A11, strain Dresden (AF296096); PTV-A12, strain CC25 (JN859128); PTV-A13, strain wild boar/WB2C-TV/2011/HUN (JQ429405); PTV-A14, strain JPN/MoI2-2-2/2015/G (LC386160); PTV-A15, strain JPN/Ishi-Ta2/2016/G (LC386157); PTV-A16, strain PX5 (QHX40812); PTV-A17, strain YC6 (QHX40822); PTV-A18, strain PX3 (MN094602); PTV-A23, strain YC23 (MN094631). PTV-A Gi2020_1 and PTV-A11 are indicated by blue arrows. Incomplete VP1 sequences are marked with an asterisk. Branch labels indicate bootstrap support values (%) based on 1,000 replicates. The scale bar represents the number of amino acid substitutions per site.

In addition to completing the genome architecture, we characterized a recent field isolate (PTV-A Gi2020_1) that shows substantial genetic divergence from established serotypes and belongs to the less virulent group of Talfan disease inducing PTVs. With only about 84% nucleotide identity to its closest relatives and a distinct phylogenetic placement, this virus likely represents a previously unrecognized genotype. The absence of clear recombination signals and its relationship to recently described strains from Asia suggest ongoing diversification within the PTV-A species. Additional phylogenetic analyses of the VP1 sequences of PTV-A Gi2020_1 with reference sequences demonstrate a notable relation to the recently proposed serotype PTV-A23 (Fig. 10). However, as demonstrated in this phylogenetic analysis, there are also closer relationships between the VP1 sequences of other serotypes, such as PTV-A1 and PTV-A11. Although VP1 is undoubtedly the most variable structural protein of PTV, comparisons of VP2 have been recommended for phylogenetic studies and typing but also failed to reveal clear correlations between serotype and genotype (71). A definitive serotype assignment of PTV-A Gi2020_1 will require cross-neutralization studies, which were beyond the scope of this work (72).

Despite this genetic divergence, the overall genome organization and polyprotein processing of PTV-A Gi2020_1 remained consistent with established teschovirus principles. Existing knowledge on polyprotein processing, virion structure, and properties, together with the availability of established serological reagents, greatly facilitated this study (5, 60, 67). Our experimental analyses of purified PTV-A Gi2020_1 virions confirmed the identity and processing of the major capsid proteins and supported the predicted 3C protease cleavage pattern of PTVs. These findings indicate that the structural protein processing is conserved—even across very divergent PTV-A genotypes. Another major advance of this study is the establishment of reverse genetic systems for two PTV strains. Completion of the 5′ end enabled recovery of fully infectious recombinant viruses that closely recapitulate the replication kinetics and growth properties of their parental counterparts. These systems provide a defined genetic framework for mechanistic studies and overcome the limitations associated with heterogeneous virus populations and passaged isolates. Infectious virus clones are valuable tools in modern virology research that have improved our understanding of positive-strand RNA viruses and revolutionized the development of vaccines and antiviral drugs. The discovery of reverse transcriptase and the generation of the first cDNA clones rapidly changed RNA virus research, as technical hurdles associated with the direct manipulation of viral RNA had severely limited genetic manipulation. In the early 1980s, the complete cDNA of the PV encoded on a pBR322 plasmid was transfected in cultured cells for the first time and was able to initiate the replication of a clonal virus (73). Subsequent incorporation of bacteriophage promoters and defined 3′-end restriction sites in such plasmids enabled *in vitro* synthesis of infectious viral RNAs (74). This paved the road for the generation of infectious cDNA clones across a broad spectrum of positive-strand RNA viruses and marked a conceptual shift in virology. Labor-intensive procedures such as the phenotyping of clinical isolates, adaptation to cell cultures, biological cloning, preservation, and conventional mutagenesis methods had, until then, hindered a rapid understanding of viral biology. Reverse genetics shifted research to work on precisely defined viral genotypes that were known at nucleotide resolution and could be genetically manipulated as desired. This enabled a direct path from viral genotypes to phenotypes, even without available cell culture systems, leading to a steady increase in knowledge (75). While naturally occurring RNA viruses are genetically dynamic quasi-species that change over time and rapidly adapt to cell cultures, infectious cDNA clones distill this diversity to a single genotype providing a conserved starting point for research studies. However, clonal RNA viruses quickly diversify again during cultivation due to the error-prone polymerase, thereby restoring natural diversity and adaptability. Reverse genetic platforms further enable the construction of reporter viruses expressing luciferases or fluorescent proteins, which have become indispensable tools for indirect virus diagnostics using neutralization assays as well as high-throughput screenings (76, 77). Finally, reverse genetic approaches enable rational vaccine design, including targeted attenuation through the deletion of non-essential virulence determinants, the use of viral vectors, and the development of DIVA vaccines (differentiating infected from vaccinated animals) (78–80). Such DIVA vaccines with negative serological markers have made it possible to distinguish vaccinated livestock from naturally infected animals and are therefore crucial for disease eradication and safe trade in livestock (81).

Using the new PTV platform, we characterized the role of the teschovirus leader protein in replication. The deletion of the leader yielded replication-competent but growth-attenuated viruses, demonstrating that the leader is dispensable for genome replication but contributes to efficient virus production. Since leaderless PTV was much more sensitive to pretreatment of host cells with IFN-α2a, we suspect that it plays an important role in counteracting the cell’s innate immune response. This phenotype is consistent with observations in other picornaviruses, where leader proteins often act as modulators of the host’s innate immune response rather than essential replication factors (37). The stability of the attenuated phenotype further suggests that the leader represents a potential target for rational attenuation strategies. To further dissect viral replication, we generated a subgenomic replicon lacking the structural protein region. This construct supported RNA replication as evidenced by reporter protein expression and dsRNA detection but failed to produce infectious particles. It was not rescued by superinfection with a helper virus, indicating that genome encapsidation is tightly regulated or spatially separated. Such replicon systems provide a valuable tool to study replication independently of virus spread. PTV replicons may facilitate future applications in antiviral screening or vaccine development removing biosafety constraints while providing a high-fidelity platform for the analysis of nonstructural protein functions. To enable direct visualization and quantitative assessment of infection, we established two fluorescent reporter virus systems. Quantitative fluorescence intensity analyses demonstrated that reporter fluorescence accurately reflected viral antigen expression and infection foci (Fig. S11). Although a split-fluorescence approach allowed for the detection of infected cells, it significantly impaired viral fitness, thereby limiting its practical applicability. The exact cause of the significant growth inhibition of this viral construct remains unclear although the size of the insert is unlikely to have played a role. We can only speculate that the insertion site downstream of 2A is unsuitable, that a 2A duplication interferes with viral processing, or that the mCherry11-SpyTag-2A fusion protein has dominant negative effects on replication. In contrast, insertion of a full-length fluorescent protein at the P1-P2 junction produced a robust and genetically stable reporter virus with only moderate attenuation. This finding demonstrates that the PTV genome can tolerate significant insertions. The reporter virus provides a versatile platform for functional assays, including neutralization tests and antiviral studies, as evidenced by preliminary experiments using a rabbit antiserum. Limitations regarding genome size for the integration of foreign genes, as well as issues with the genetic stability of reporter viruses, as observed in related picornaviruses (e.g. EMCV and PV), seem to pose fewer challenges with PTV-A11 (82, 83). Since several stable reporter viruses for picornavirus-like pathogens have already been successfully developed in the past, species-specific restrictions and limitations on insertion sites must be assumed (84).

Together, our study provides new mechanistic insights into the genome organization, the function of the leader protein, and the replicase of teschoviruses. Furthermore, the molecular clones establish a foundation for future studies on PTV replication, pathogenesis, and immune control. In the context of ongoing viral emergence in swine populations, such tools are crucial for improving preparedness and developing targeted intervention strategies.

## Data Availability

The complete genome sequences of PTV-A11 (strain Dresden) and PTV-A Gi2020_1 have been deposited in GenBank under accession numbers AF296096.1 and MW824925, respectively. All additional data generated or analyzed during this study are included in this published article and its supplemental material. Further information is available from the corresponding author upon request.

## SUPPLEMENTAL MATERIAL

Fig. S1 to S5 jvi.01036-26-s0001.pdf Immunofluorescence analyses, genome sequences, and RNA secondary structure predictions.

Fig. S6 and S7 jvi.01036-26-s0002.pdf Immunofluorescence analyses, RNA secondary structure predictions, virus characterization, and CsCl gradient purification.

Fig. S8 to S10 jvi.01036-26-s0003.pdf Immunofluorescence analyses, interferon sensitivity assays, split-mCherry reporter analyses, and serum neutralization experiments.

Fig. S11 jvi.01036-26-s0004.pdf Fluorescence intensity profile analysis of viral antigen and reporter protein expression.

ASM does not own the copyrights to Supplemental Material that may be linked to, or accessed through, an article. The authors have granted ASM a non-exclusive, world-wide license to publish the Supplemental Material files. Please contact the corresponding author directly for reuse.
