# Supplementary material for: Completion of the 5′ end of the porcine teschovirus genome reveals a functional S-segment and enables reverse genetics
Source: J Virol. 2026 Aug 11;100(9):e01036-26. doi: 10.1128/jvi.01036-26 (PMC13595913; doi:10.1128/jvi.01036-26)
Supplement: Supplementary file 1 [file jvi.01036-26-s0001.pdf]

Part 1 Supplemental Figures and Legends for „Completion of the 5′ End of the Porcine Teschovirus Genome Reveals a Functional S Segment and Enables Reverse Genetics"

Sebastian Affeldt[1], Sandra Barth[1], Stephanie Schlimmbach[1], Irmin Lobedank[1], Josef Kuehling[2], Thomas Timm[3§], Günter Lochnit[3], Sabrina Becker[2], Enrica Sozzi[4], Sven Reiche[5], Friedemann Weber[1], Gerald Reiner[2], and Benjamin Lamp[1*]

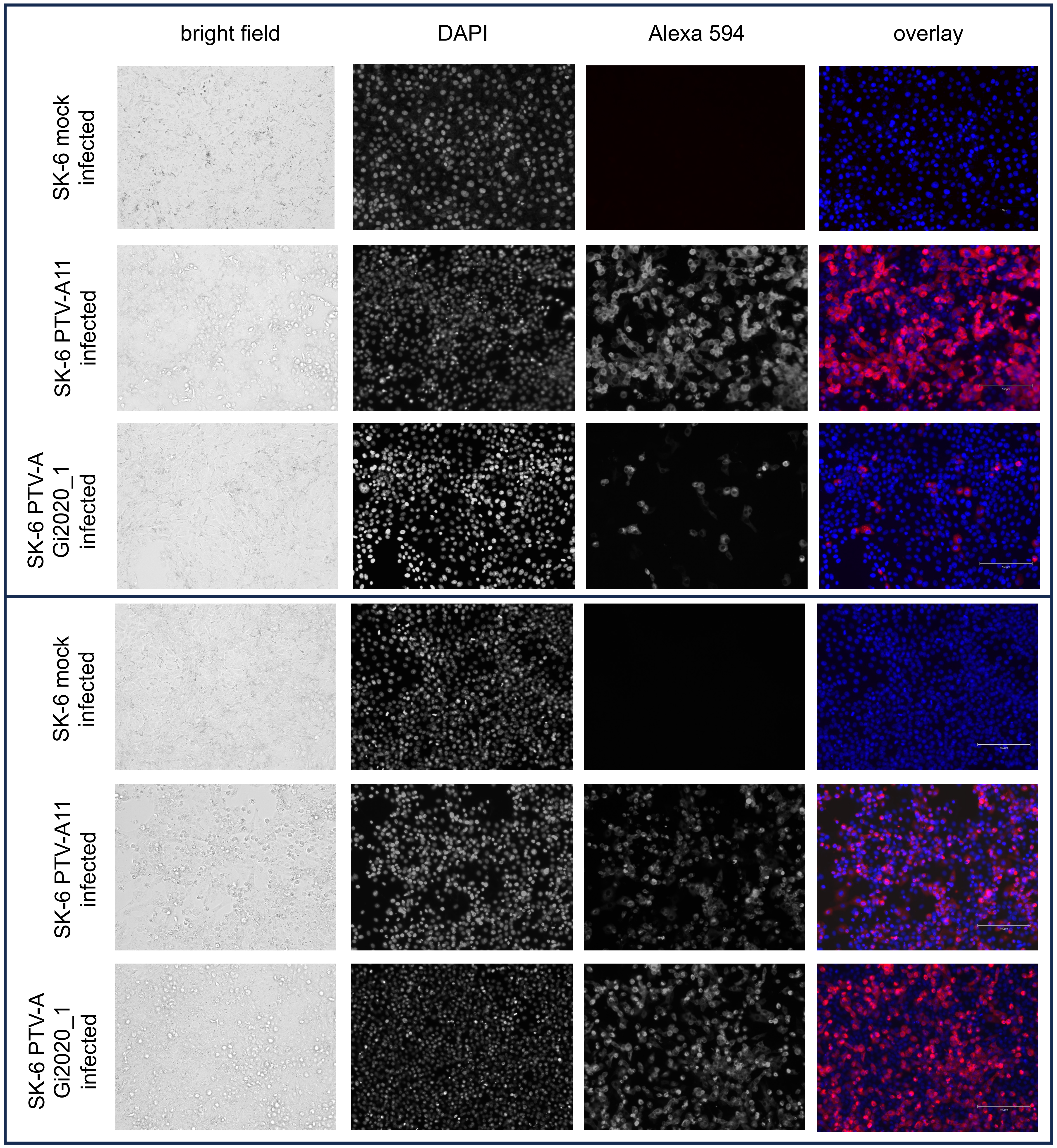

Figure S1. Indirect immunofluorescence analysis of SK-6 cells infected with PTV-A11 (strain Dresden) and PTV-A Gi2020_1. Cells were infected at a MOI of 0.01 and fixed at the indicated time points. (A) At 24 hpi, PTV-A11 infected approximately 50% of cells, whereas PTV-A Gi2020_1 showed limited spread with approximately 1% antigen-positive cells, occasionally appearing as doublets. (B) At 48 hpi, the PTV-A11-infected monolayer exhibited pronounced cytopathic effects and reduced fluorescence intensity, whereas PTV-A Gi2020_1 had spread to approximately one-third of the cells. Infected cells were stained with the PTV-specific monoclonal antibody 4B1 and an Alexa Fluor 594-conjugated goat anti-mouse secondary antibody. Nuclei were counterstained with DAPI. Representative overlay images are shown. Scale bar, 150 µm with original magnification of 20x.

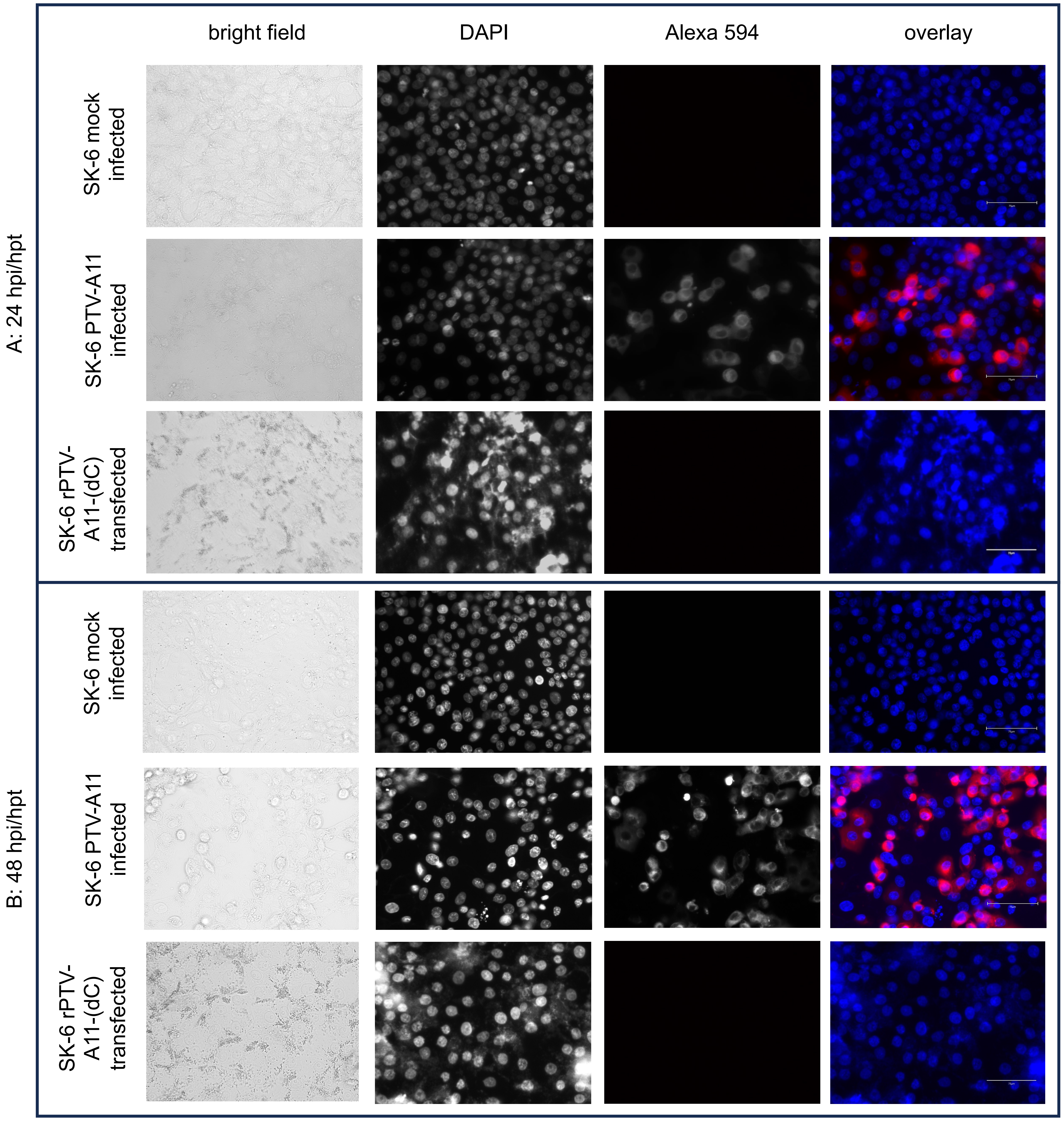

Figure S2. Absence of viral protein expression following transfection of rPTV-A11-(dC) RNA. Indirect immunofluorescence analysis of SK-6 cells infected with PTV-A11 (strain Dresden; MOI = 0.01) or transfected with in vitro-transcribed rPTV-A11-(dC) RNA. (A) At 24 hpi/hpt, PTV-A11-infected cells displayed the characteristic cytoplasmic staining pattern of PTV capsid proteins, whereas no viral antigen was detected in cells transfected with rPTV-A11-(dC) RNA. (B) At 48 hpi/hpt, PTV-A11 infected cells exhibited pronounced cytopathic effects while remaining antigen positive. In contrast, no viral antigen was detected in rPTV-A11-(dC)-transfected cells. Cells were stained with the PTV-specific monoclonal antibody 4B1 and an Alexa Fluor 594-conjugated goat anti-mouse secondary antibody. Nuclei were counterstained with DAPI. Representative overlay images are shown. Scale bar, 75 µm with original magnification of 40x.

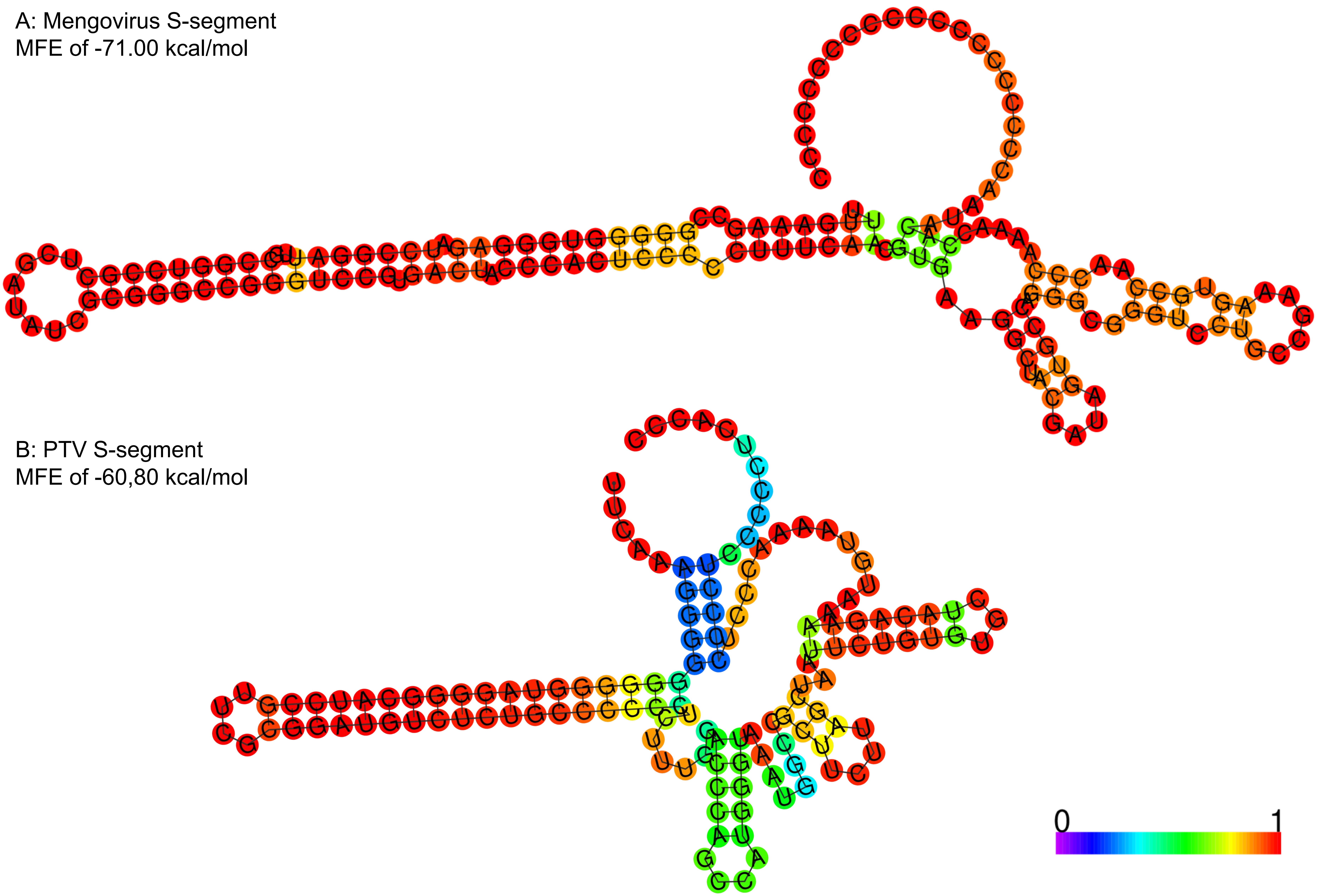

Figure S3. Predicted secondary RNA structures of the S-segments of mengovirus and PTV-A11. (A) Predicted secondary structure of the 147-nt S-segment and adjacent 20-nt poly(C) tract of mengovirus. The established stem-loop structure spanning nts 3–84 exhibits a high base-pairing probability, whereas the poly(C) tract is predicted to remain unpaired. (B) Predicted secondary structure of the 118-nt S-segment and adjacent 20-nt poly(C) tract of PTV-A11. A stable stem-loop structure is predicted between nts 11 and 50, with a high base-pairing probability located in the apical region. In contrast, the 5'-terminal nucleotides and the poly(C) tract are predicted to remain largely unpaired. Structures were predicted and visualized using RNAfold without folding constraints. The average minimum free energy (MFE; kcal/mol) is indicated above each structure. Nucleotides are colored according to base-pairing probability as shown in the scale below. For unpaired nucleotides, the color indicates the probability of remaining unpaired.

rPTV-A11 (strain Dresden)

```
TTCAAAGGGGGGGGGTAGGGGCATCCGTTCGCGGATGTCTCTGCCCCCCTCTTTGGACCCAGCCATGGGTAAATGGCCGCTTCTTAGCTAATTCTGTGTGCTACAGAAAATGTAAAACCCTCCCCTCCCCCTCACCC
TTTGAACTTGTAACTGATAAGTGATGATCCTTGGAAGCTAGGTACTCGTTACGCTAGTTTTGGATTGTCTTGTGCCCAACACTAGACCTCGGTCTCTTGTTGTGTTTAAACACAGAAATCTTGTTATTTGGTTATGA
ATTCATTGGATCAATCCCTTTGAAAGACCTGCTCTGGCGCGAGCAAAAGCGCAATTGTCACCAGGTATTGCACCAATGGTGGCGACAGGGTACAGAAGAGCAAGTACTCCTGACTGGGCAATGGGACTGCGTTGCAT
ATCCCTAGGCACCTATTGAGATTTCTCTGGGGCCCACCAGCATGGAGTTCCTGTATGGGAATGCAGGACTGGACTTGTGCTGCCTGACAGGGTCGCGGCTGGCCGTCTGTACTTTGTATAGTCAGTTGAAACTCACC
ATGGAATGTTTAAAAATTTTCTCTCTGAAACCCAAAAGCAAGAGCCACTCATACTCTCCACGTGGAGTTGAGTTGAAATATAATTCAGATTTTGCTTTTAAGCCAAGACCACTAGCACCCCTCTTGCGTCTTGAAAC
ATCTGATATCAAAACGCGCAGGATTGAGTGCGCTAATGTGGAATATGATTCATGGTACCCTAACCCTTTGGACCCAACTTCCTTAGCTTGTGATGTGGATCTTGAGATGCTACAGTTCCAAGGTACTGGATCCTCCA
GGATGGAGAATGGAAATACAAACAACTCTGGTAATACTGGAGTGATCAACTACAACTTTTATTCCAATTCTTACACAGATGCAATTGATCTCTCCGGAGCAATGTCATCACAAGAGTCAAATGCAGCTGAAAATGCT
GCGTCAGGCCCCTCATCTTTGCTTAAAGCAGGTATAAATGCTGCTGCTAGAATAGGGCCACTGCTTGCGGAGCCCAAATTTGAGGAATACAAGCTATCAGATAGAGTTGATATGCTTCAAAAAGGGACTAGCACAAT
AATTTCACAGAATAGTGTGGGATCTAATACTTATGTTGATTGTCCAAATTTTGATGTGACATCAGTTGCAGATGAGGGAACATCTGGTGGTCCAGCAGTGTCTCGGTTTGTGACTCTTAAAGCCCAGCCGTGGTCAA
CATCGCAAGATGTATATTCATTTCAAGCTGCCCACTTGCCCTATGCTTTGGTTTCACAGGAAACGCCATTTAAGGCTCTTTTGTCTAAGCACCAGCTACTTAAATGTGGTTGGATAGTCCAAGTGCAAATCAATACA
ACCAGATTCCACGGTGGCTGTATCGGTGTGTTTGCTGTTCCTGAGTTCAGTGTCTTTTCCCGACCTGCTAGCAGTCCACCCACACATTCTAACAATAGTGGTGGTTCCCCAAAAGATATTTGGGACAAGTTCTCACA
ATGGCATGACCCGAACAAAATGTATAGCACTTGGTATTATCACCATGTTTATGACAAAGATGACAAGCACTGGTATAAACCAGAAGAGCAGCCGTATGGCGGGATATCACCCCAGTCTCTGTTTTGCTTCCCCCACC
AGCTAATCAATCCCAGGACAAACTCATCAGCAACACTGTGTGTGCCCTATGTGGATTGTGGTCCTATTACTGATGTCACAGTCCACTGCCCCTGGGCTATTGTAATTGTGGTTTTGCGCCAGCTATTAGTAGCTAAC
GGTGGGACACCTAGTGTTGACATCAATGTTTCTTTGGCACCCTGCAATGTGGAATATCACGGGCTTCGTCAAAGTTCTGTGTACCAAAGTCCAATACCAAAAGTTGATCTTGATGCTAGCAGGGCATTGTTTTCTTC
AACTCAACCATATACAGCTGCCCCCGTTTATGGTAGGATCCCTGCTAGTAGCCCATCCTACATGCCAGGAGAATTCCTTGATTATGTCCAACTTGCTCAAATTCCCTCTTTGGTGAAGAATCGGGAAATTCGTGTGT
CCAATTCAGAAAAGACCAAGCCAATTTTTGGGATGCCTTTGAGACCAGTTAATGAAGAAACCACAAACACCATGTTAGGGTCTGTTACAAGGATGTTTACACAGTGGCGTGGTTCTATAGTTATAAGGTGTGTGTTT
GTTGGGAATCAGATGCAGAATTGCAGACTTGTTTTAGCCTGGACACCACAGGGCTTTAATGCACAAGAGCCAACAAGCATGGCTGAGGCAATGCAAGGACACTATGTCATCTATGATACTGGTGTGGACAGTAGCGT
GGACTTGGTTGTTCCTTACGTAGCACCATATGATTTCACGCCCGTGCGGACTCCCTACACCAAGACAGACTTTCCTATGCAGCCAAATAGCAATCCCGTTTTGGTTGACACACCATATTGGTCCAAGGGATATGTGA
CAATCTGGCAGTATACTAATCTGGCATCTCCCCCCAATTCACCATCCCAGGCTGATTTTTTATTGTTTGTTTTTGCCGGGAAAGACTATGTGTTAAAGGGACCCTCAAACATCTCTTCTGGCTTTCAGGGAAATGCT
GACTGTCCACTTCAGCCGGCTGAGACAGGATGTGACAAAGCAATGACAGCTAGTGCATCAAATGAAAATCCCATTCCCTTGGCAGTTGAAAACATCGGTTCATCCCTGGTCTCATTTTTCTGGGACAGGTACTTCCA
CATTTCAACAATTAGTAAGATGACTAATGTTGATCCACAGTATTTGCGGTTGAACATCTCAGACATCTTGCTGGATTCTGTCATACGCCAGTCTTTGCACGCCACCTATTTGAGATGTGGTTTATCTGTTGCTGTGA
GAGTGATGCCAGCGAACCCTGCTAGCAATGAATGCCTGGATGGTTTGACAGTGAATTTATTATATGTCCCTCCTGGGAGTGGTTGGAACATTAATAATGTTGATGCAACAGCCCCCATTGAGCCAGCTAAAGATTCA
GTGGACAATTATGCATTACCCTCATTCACATGGAAACCAGCGCAGACACCGGTTTTTACGTGTTCAGTGCCATACGTTTCTTTCACCTCTGTTCTGCCCACCATGTATTCTGGCTTTGAATCATCATCAAAAATCCC
AAAAAGAAATAATCAGATTCCCCAAGACTTTGGGTTTGGCATGCTTGTGCTTAGATCCTCGTCTACATTGGGATTAACAGCTTCTGTGTGGGTTAAATTTGAAAATGTGAGACTCTTCTGCCCACGGCCTGGGATTC
ATGATCCTTTAGCCTATTCTAGAAGCCATCCTCAGGAAAATAGAAAGTATGACGGAGACCAATTGTCAACTGACATGACCAGGATGTCATTTCAAGGGCCGGGTGCCACAAACTTCTCACTCCTAAAACGAGCAGGG
GATGTTGAAGAAAATCCCGGGCCCACCCTTTCTAAATTGTATCAGATTTTGAAAGACCCAGCAGTTGATGCTTTGTGTGGAGCATATGATGAATTGAAGAAATTTAAAGAGCAAGCTACTAACTTGCTCGACTCATT
TTCTGGGGATTCTGAAAATCCTTGGTTAAACAAATTTATTAAATATCTTGGGTATGCCATCCTGGCATGGAAATCCCTCCATGACCCAATGACAGCAGCGGCAGTTTGTTTTATAATTGGCAGTGATGTGACAGCAT
TTGTTGTTTCCAAATTGGCCAAGCATTTGAAGAGATTTGCGAAAACAGATCCACCCCCTGTGCCTAAACCTAGATCCTGCAAGAAAGAAGGATGTTGTTGTGGTAACAAACACAACTACCCCGATGAGCTTAATCCA
TTTTCTGAAAATGGTTTCTGGAGCCGCTTTAAGAAAGGCCACCTCCAGGGCCCCATGCAAGACGTGAGCTCTCTAATTAATATTTTGAAGGGTGCAGAATGGATTTATCACCAATTTGAGAAAGTGATTAAATGGCT
TAAGACCTGGAGAACTGCTGAATATGAGGTTTCTCCTGATTTTCTTCAAGCTCAGATGAAGGAGTACCCACACTACTACAGGAAATACAAGGAGTGTGCTAACAACTATAGGCACCCAGATAGGACTGAAGTGGCCA
ACTATTTTAAGAAGATGAGGAAGTGTGCTGCTTATGTGAACCCAAGGTTAATGGGTATGTTCCCTGAATTTGACCCATCTCCACCTGATCCAACTCGCCCAGAGCCGGTAGTTGTTGTTCTCCGCGGTGCTCCTGGT
CAAGGGAAAAGTGTGTGCTCTGAAATGCTTGCTAGAATGCTATCCTATGTTTTAGTTGGCAAATCCTCTTATTATGCTTTTAATTCAGCTACTAAGCATTTTGACGGCTATAAACAACAAGCTGTCACAATAATTGA
TGACCTTGGGCAAGATACATCCGGTGAAGACTTCCGGCTGTTTTGTCAGCTAGTGTCCACTACTGAATGTATTGTGCCTATGGCTGATTTGCCTGATAAGGGAATGCATTTTAAATCTGAAATTCTTATTGCTAATA
CCAATTTGCCTTCCTTTAACCCTATCACTGTATCTGATCCAGCTGCAATAAAGAGGAGAATTTTTCTTGATCTTACAGTTGAAGCAAATCGGGCCTATACTAAGCCTGATGGGACACTCGACCTTGTCCGAGCGCTC
CAATCAACTGGGAAGGAATCTCAGTCTCCACTTTTAGACAGGACCATCACATCCTTTATTCAGATTGTGTTGCCTTTAAGCATGGTCGACACACCCTGTCATTCTTGGAAGTGTTCGACATGATCAAGAGAGAACT
ACAGAGACGCCAAAATGTCTCCAATTCTTTGACAAATCTCTTTACTTTCCAAGGGCCCAGTGATGACTCCTGGTTTACCACTTTTTACAAGAAGTGGAATCTGAGAGCAAAAGAAAACTCTGAGGAAAAACTGATCC
TGGAGCTTATCAGGTACTGTCATGGATCTGATATGCTAAAGGAATACTGCCAGCTGGCAGCAGCCAATAAAGAAAAGAAAACAGACTATTGGAACATGGTTAGGATAATTGAAGAGATTCTTGCAGCCTTGACTTTG
ATTCTGTCCCTCATTTCCATCATGATAGTGATGTATCAACTCTTTTTCCAGGGGCCCTATGAGGCTAGCGCAGTAAAACCCGCAAAGCCAAATAGACAATCTCTTCTTAAACTTGTTGAGATGCAAGGGCCAAAAGG
GCAAGCCAACATGGAAATGGAAAGGACCCTGATGAAAAAGAACATTGTTGAGATGACCTATGAGAAGCACAATGGGAAATTTCAGACAACTACTGTCTTGTTTGTTTGTGAGCGGATCTTCCTGATTAACACCCATA
TATTAACATCAATTAAGAACTTCCACTATGAGAACACTGAGATACCAGCAGCCTCTGTTCAGAAAGTGCAGGCTATCTTTGATGGGCACCCGTCTGATGTCACAGCAGTCAAATTTACTGTTGGACGCCAGTATCGA
GATATCAAGGACAACTTCATTAACAGCCTGCCAAACCCTGGAACACCCATAGTTGGGTTGATGAAGACAGAAGGCAGCAGCTATATGTGGTCTGGTGAGTGTCTTCCATTTAAGAACACAATGAACACCTATGAAGG
TTGTGTTCCACATGTGCTTCCTTACAAAGCTGTAACAGCCCATGGTTATTGTGGTTCTGTGATGGTTGCAGATGCTGGAGTCTGGAAAGGCATTTGTGGCATCCACTCCCTCGGAGATGGCGCCATAGGTGCAGCAA
CTGTATTGTCAAGACAGCATTTGCTCAACCTTCTTGAGGGATTTCTGGAATTTCAAGGAAAGATCTATGATGTGCAGAAAGCTGACTTTGTGTACACACCTACCAAGACTTCTTTGAAACCTACATTTGTGTGTGTT
GATCCAAAGTTGGAGCCAGCAGCCCTTTCACACTTGGACCCAAGATTGAAAGAACCAGAAAATTTTAAAGCTGTAATTCTGTCCAAGCATGTGGGTGATTCACTGCAACTGCCATGGGGCATTCGATGGGCCTCGTA
CAAGTATGCAGAGAAAATACGTGCCATGCTACCACCGGACTTCCTTGAGCCTCTTTCTGTGAGGGAAGCAGTTGAAGGAATTGATGGACTGGATCCAATAGATATGGATAAATCGCCTGGACTTCCATATGTCAAGA
AAGGCTTGCGTCGGACTGCCCTGTGGAACCCCAAGACTGGACCTTCACTGGAACTGATGGCTGAGATAAACCGTTATCTTGACTACAATTATGATAAACACGTGTTTCTGACATTTTTAAAAGATGAACTGAGACCA
AAAGAGAAAGTTCAAGCTGGGAAAACCCGTGTCATTGATGTAGCAGGGTTTGGGCACGCAATAGTCGGCCGGATGCTATTCGGTCGGCTGTTTGCCTTCTTTCATAAGAACCCCGGTTGGAACACAGGATCAGCTGT
TGGAGTTAACCCGGACTTGGCCTGGACTCAAATCTTTTACACAGCCCCCAGCCGTAACGTGCTGGCTATGGATTATTCTGGATTTGATGCCTCTCACACATCTGGCATGTTTTGCATTTTGAAACATTTCCTTACCA
CTCTTGGTTATGGAACTTTACAGCTATCCTATATTGATTCTTTGTGCTATTCTAAGCACCATTGGGATGATGAGACTTACCGCCTTGACGGTGGATTGCCCTCAGGATGTTCGGGCACAACAATATTTAATACAATT
ATGAACAACATTGTTGCCCGTGCAGCAGCCAGCTACGCTGCCGATGGCCCTGTTGGCATCCTCTGTTATGGTGATGACATCCTTGTCTCATCTCCTGAAAAATTTCCAGTTTCTGACTGGCTGGAGTTTTACTCCAA
GACACCTTACAAAGTGACTGCAGCTGATAAGAGTGAGCAAATTGACTGGCGCGACATCACACAGTGCACGTTCCTTAAACGGGGATTTGTGCTGGATGGTAGTCTTGTGCGTCCAGTTATGGAAGAACAACATTTGG
CTGAATTGCTGAAATGGGCTAGACCTGGAACATTGCAAGCCAAGTTATTATCCATTGCCCAATTGGCTTTTCATCTTCCACGCCAAGCCTACAATCGCTTGATGCTCCCGTTTGAGGAGGCTGGGTACGAAATACCT
AGCCACGAACGCCTCAATGAGGAGTGGCATGAAATGTTCATTTGAACAGCTACAGTTGTGAGCAGTTTAGTGATTTTCTGCCTGTTGCCCAAGTAGTTTAGTTGAGC
```

Figure S4. Genomic sequence of the rPTV-A11 clone. In this study, an infectious molecular clone of PTV-A11 (strain Dresden) was generated using a pBR322 system. The complete sequence, including the previously unknown S-segment of the 5'-UTR excluding the poly(A) tail, is presented here. A reference sequence for the strain has already been published under AF296096.1. The functional clone rPTV-A11 differs from the reference sequence - excluding the poly(C) tract and the novel S-segment of the 5'-UTR - by only six nucleotide mismatches and an insertion of three nucleotides. These nucleotide differences resulted in four amino acid differences including the homologous exchanges K208E, Q339K, and S2817N as well as a serine insertion (D2022L to D2022SL). A serine residue at this position is highly conserved among other PTV strains. The PTV-A11 genome is relatively AU-rich (55,43%) with 27.2% A, 28.3% U, 22.3% G, and 22.2% C. The individual regions of the genome are annotated and labeled as 5'-UTR (blue), Leader (black), VP4 (sky blue), VP2 (almond), VP3 (green), VP1 (lemon), 2A (orange), 2B (red), 2C (pink), 3A (bold blue), 3B (bold green), 3C (bold red), 3D including stop codon (bold blueberry), 3'-UTR (bold black).

rPTV-A (strain Gi2020_1)

TTCAAAGGGGGGGGGTAGGGGCATCCGTTCGCGGATGTCTCTGCCCCCCTCCTTGGACCCAGCCATGGGTAAATGGCCGCTTCTTAGCCAATTCTGTGTGTTACAGAAAATGCAAAAACTCCCCCTCCCTCTCCCCC
CCACCTCCTTTGAACTTGTAACTGATAAGTGATAGTCCTTGGAAGCTAGGTACTTGTTACGCTAGTTTTGGATTATCTTGTGCCCAGCACTAGACCTCGGTCTCTTGTTGTGTTTAAACACAGAAATCTTGTTATTT
GGTTATGAATTCATTGGATCAATCCCTTTGAAAGACCTGCTCTGGCGCGAGCAAAAGCGCAATTGTCACCAGGTATTGCACCAATGGTGGCGACAGGGTACAGAAGAGCAAGTACTCCTGACTGGGCAATGGGACTG
CATTGCATATCCCTAGGCACCTATTGAGATTTCTCTGGGGCCCACCAGCGTGGAGTTCCTGCATGGGAATGCAGGACTGGACTTGTGCTGCCTGACAGGGTCGCGGCTGGCCGTCTGTACTTTGTATAGTCAGTTGA
AACTCACCATGGCATGTTTGAAAATTTTCTCTCTGAAACGTAAAGATAAGAGCCACTCATACTCTCCTCGTGGGGTTGAGTTGAAATACAACTCAGACTTTGCATTTAAACCAAGACCACTAGCACCACTTTTGCGC
CTCGAACCATCTGACACCAAGACGCGCAGAATTGAGTGTGCTGAAATTGAATATGACTCATGGTACCCAAATCCTTTAAATCCAACTTCTTTAGTTTGTGATGTTGAACTTGAACTGCTGCAGTTTCAAGGGACTGG
GACTTCTAGAATGGAAAACGGTAACACCAACAACTCTGGTAACACTGGTGTGATTAATTACAATTTCTATTCTAACTCATATACTGATGCAATTGACCTTTCTGGGGCTATGTCCTCACAAGAGTCAAACGCTGCTG
AGAATGCTGCATCAGGACCCACATCTCTACTCAAAGCTGGCATAAATGCTGCTGCAAAAATTGGACCCCTACTTGCAGAGCCCAGATTTGAGGAATACAGGTTATCTGATAGAGTTGATATAATTCAAAAAGGCACC
AGCACAATTATATCACAAAACAGTGTTGGATCTAACACTTATACAGACTGTCCAAAATCTGAAGTGACTTCTGTAGCTGATGAGAGTACGTCTGGTGGACCAGCTGTTTCACGGTTCATGACTGTGGAAGCCCTGCC
TTGGACTGCTGCACACGGTGTGTATTCATTCCAAGCTGCACATCTTCCATATGCACTGATAAAGCAAAACACTCCATTCAAAGCTCTTTCATCAAGGCACCAGCTTTTAAAATGTGGATGGATAGTGCAAGTACAGG
TGAATACAACTAGATTTCATGGTGGATGTCTTGGTGTTTTTGCCATTCCTGAGTTTAGTGTCCATTCAAAGCCAGATAATACAACACCAACATATGATAACAACATTAACGGAGCTGTTACTGATATTTGGGACAGC
TTTACAAAATGGCATAATCCCGACAAAATGTACGGAACATGGCACTACACCAATGCTTATGATCAAACTAGAGACAAACACTGGTATAGACCACTTGCGCAACAATATGGTGGACTTTCCCCCCAATCATTATTTTG
CTTTCCTCACCAACTAATAAACCCCAGAACAAATTCTTCTGCCACCCTGTGTGTACCATATGCAGATTGTGGCCCAATAACTGATATTACAGTGCACTGCCCGTGGGCTATTGTAATTGTTGTTCTCAGACCACTTA
CAGTAGCCAGTGGAGGTACAACAAGTGTGGATATAAATGTTTCTGTGGCTCCTTGTGAAGTGCAATATCATGGGCTACGTCAAGATTCTATGTATGAAGGACCTGTTCCCAAAATTGATGTTGATGCCAGTATGGCA
CTTTTTTCATCCACCCAGCCTTATCAAGCACCTCCTGTTTATGGCAAAATTCCAGCAAGTGACCCCTCTTTTGTCCCTGGAGAATTTAGTGACTATGTTCAGCTTGCTCAAATTCCAAGTCTTGTGAAGGACCGCGA
AATCCGCGTTTCCAGTAACAACCAGACAGAGCCTATTTTCACCATGCCTTTGAGACCAGTGAATATAGAGACAACGAATACCATGCTGGGATCAGTCACTAGGATGTTTTCACAGTGGCGCGGATCAATAGTTGTAC
GGTGTGTCTTTGTAGGCAATCAAATGCAGAATTGCAGGCTTCTTTTATCATGGACACCAGAAGGCTTTTCAGCAATACCGCCCAGAACTATGGCACAAGCAATGCAAGGACATTATGTTATCTATGATACAGGGGTT
GATAGTAGCGTGGATTTGGTAATACCCTACGTATCTCCCTATGACTTCACCCCTGTTAGAACACCTTACACAAGGACTGAGATCCCACCAGTGCAAACCACACAGAAAGTTTTGGTTAACACCCCCTATTGGTCAAA
GGGATATGTTACTGTGTGGCAGTACACCAACCTGGCATCCCCCCCCGGCTCACCATCACAGGCTGATTTTCTGTTGTTTGTGTTTGCTGGGAAGGACTATGTCTTGAAAGGCCCATCAAACATTGCAGCCAGCTATC
AGGGCAATGCTGACTGCCCCCTTCAGCCTGCAGAAACAGGTTGTGACAAGGCACAAACAAATGAGGCCTCTAATGAGAACCCAATCCAGTTAGCAGTGGAAAGCATAGGGTCCTCAGAAGTGAGATTTTTCTGGAAT
AGGTATTTCCATGTTGTAACAATAAATGGCACCTCAAACACTGCCCCACAATATTTGAGATTAAATATCGCAGATATCTTGGCTGATTCTGTGATTAGGCAGTCACTACACGCAACTTACATGCGATGTGGTCTTTC
CATTGCTGTTAGAATTATGCCCAACAGCACATCAGATGCCATCAATTTGGACTGCCTTACAATTAATATGACATATGTCCCCCCTGGTAGCGGCTGGGCCATTGATAACATCGTGGCGAGTGCCCCCATTGAAACGG
CCCAAGATTCCACAAAGAATTATGCACTGCCATCTTTCACGTGGAAGCCCAGTCAAACACCTGTTTTTACATGCTCTGTTCCATATGTTTCTTTCACATCTGTATTACCAACTGCTTATTCTGGGATTGAGACACCT
TCCTTAATACCAAAGAGGAACAACCAGATACCCCAGGATTTTGGGTTTGGCATGCTTATTCTGCGGCCTTCAATGCAAATTAGTTTGGTTGCATCACTTTGGGTGAAGTTTGATAATGTGAGGCTATTTTGCCCGCG
CCCTGGCATTCATGATGCAGAAGCCTATTCAAATGCATCTACCCTAATCCCTGAGCCGGGGACCGGCAGATTATCAACCGCCATGACAACACTGTCACTTCAAGGACCAGGTGCCACTAATTTCTCCTTGCTAAAGC
AAGCAGGAGATGTTGAAGAGAACCCAGGGCCTTCCCTTTCTAAACTATACCAAATTCTTAAAGATCCAGCAGTTGACGTATTATGTGAGGCCTATGATGAGTTGAAGAAATTTAAAGAGCAAGCTACCAACTTGTTA
GACTCATTTTCTGGAGAATCAGAGAATCCTTGGCTAAATAAGTTTGTTAAATACCTTGGGTATGCCATCTTGGCTTGGAAATCCCTCCATGACCCAATGACAGCAGCAGCAGTCTGCTTCATAATTGGTAGTGACGT
GACAGCATTTGTTGTCTCCAAATTAGCCAAGCATTTGAAGAAATTTGCCAAGACTGATCCACCTCCAGTACCCAAGCCCAGAGCCTGCAAGAAGGAAGGGTGCTGCTGTGGTAACAAGCACAACTACCCTGATGAGC
TGAACCCCTTTTCAGAGAGCGGCTTTTGGAGTCGGTTTAAGAAAGGCCATTTCCAAGGCCCAATGCAAGATGTGAGCTCTTTAATTAACATCCTGAAGGGTGCAGAATGGATCTACCATCAATTTGAGAAAGTGATC
AAATGGCTCAAGTCATGGAAAACTGCTGAGTATGAGGTTTCTTCTGGCTACTTTCAGACCCAGATGAAAGACTATCCACACTATTACAGAAAATATAAGGAATGTGCAAATAATTACAGACACCCAGACAGAACTGA
AGTGGCAAACTACTTCAGGAAAATGAGAAAGTGTGCTGCCCACGTTAATCCAAGATTAATGGGCATGTTTCCTGAATTTGACCCTTCCCCTCCTGACCCCACTCGTCCTGAACCAGTTGTGGTCGTTTTGCGCGGCG
CACCTGGACAGGGCAAGAGTGTGTGTTCTGAAATGCTTGCTAGGATGCTTTCTTATACCTTAGTTGGCAAGTCTTCTTATTATGCATTTAACTCAGCCACTAAGCACTTTGACGGCTATAAACAGCAGGCTGTCACA
ATTATTGATGATCTTGGGCAAGACACATCTGGTGACGACTTTCGGCTGTTCTGCCAACTAGTGTCAACTACTGAGTGCATTGTGCCCATGGCTGATTTGCCAGACAAAGGAATGCATTTTAAATCCGAGATACTTAT
TGCCAACACAAATCTGCCCTCTTTTAACCCCATTACTGTGTCTGACCCAGCTGCTATAAAGAGGAGAATTTTCCTTGACCTTACAGTCGAGGCAAATCGAGCTTACACCAAGCCCGATGGGACACTTGATCTTGTAC
GAGCACTGCAATCAACTGGCAAGGAATCCCAGTCCCCACTTTTTAGACAAGATCATAACATCCTTTATGCTGACTGTGTTGCCTTTAAGCATGGTAAATACACCTTATCTTTTCTGGAAGTGTTTGATATGATTAAG
AGAGAGCTGCAGAGACGCCAGAATGTTTCTAACTCTTTGACTAATCTTTTTACCTTCCAGGGGCCAAGTGATGATTCCTGGTTTACTACCTTTTATAAGAAGTGGAACCTAAGAGCAAAGGAAAATTCTGAAGAAAA
ATTGATTCTTGAGCTTATTAGGTATTGTCATGGATCTGATATGCTAAAAGAATACTGTCAGTTGGCAGCAGCCAACAAGGAAAGAAAAACAGATTATTGGAACATGGTTAAAATTATTGAGGAAATTCTTGCAGCTT
TGACCTTGATTTTGTCTCTTATTTCCATTATGATAGTGATGTATCAGCTCTTCTTCCAGGGACCCTACGAGGCCACTGCAACAAAGCCAGCAAAACCTAACAGACAATCCCTCTTAAAGCTTGTTGAAATGCAGGGA
CCAAAAGGACAAGCCAACATGGAGATGGAAAGAACCTTGATGAAGAAAAATATTGTGGAAATGACTTATGAGAAACACAACGGAAAGTTTCAAACAACCACCATTCTGTTTGTGCGCGATCGGATTTTTCTGATCAA
CACTCATATCTTAACAGGCATAAAGAATTTCCACTATGAGAACACTGAGATTCCAGCAGCCTCTGTCCAGAAAGTGCAGGCTATATTTGATGGACATCCATCTGATGTGACTGCTGTTCAGTTTACTGTGGGCCGAC
AATACCGGGATATAACGAGCAATTTCATTAACAGCTTGCCAAGCCCTGGAACACCCATAGTTGGGCTGATGAAGACTGAAGGCAGCAGCTACATGTGGTCTGGTGAATGCCTCCCATTTAAGAACACAATGAACACC
TATGAAGGTTGTGTTCCACATGTACTTCCGTACAAAGCTGTAACGGCCCATGGCTATTGCGGCTCTGTTATGGTTGCAGATGCTGGAGTTTGGAAAGGCATTTGTGGTATTCATTCTTTAGGGGATGGTGCCATAGG
TGCAGCCACTGTTTTGTCAAGACAACACCTACTTAACCTCCTTGAGGGATTTTTGGAATTTCAAGGGAAAATTTATGATGTGCAGAAGGCTGACTTTGTATACACACCAACCAAGACTTCCTTGAAGCCAACATTTG
TGTGTGTTGACCCAAAACTAGAACCAGCAGCCCTTTCACACCACGACCCAAGATTGAGAGATCCAGAAAATTTTAAAGCTGTCATTTTATCCAAGCACGTGGGCGATTCGCTGCAACTGCCTTGGGGTATTCGATGG
GCCTCATACAAGTATGCTGAGCGAATACGTGCCATGTTGCCACCAGACTTTCTTGAGCCTCTCTCTGTAAGAGAAGCAGTTGAAGGAATTGATGGATTAGACCCAATGGACATGGACAAATCACCTGGGTTGCCATA
CGTCAAGAAAGGCTTGCGCCGGACTGATTTGTGGAACCCAAAAACAGGACCTACAATAGAGTTGATGGCTGAGATAAACCGCTATCTTGATTACAATTACGATAAGCATGTGTTTTTGACATTTTTGAAAGATGAGC
TGAGACCAAAAGAGAAAGTTCAAGCTGGGAAGACTCGCGTCATTGATGTGGCAGGGTTTGGGCATGCAATAGTTGGCCGGATGCTGTTCGGCCGGCTGTTTGCCTTTTTCCACAAGAACCCCGGCTGGAACACAGGA
TCAGCTGTTGGGGTCAATCCAGATTTGGCCTGGACTCAAATCTTTTATACAGCTCCCAGCCGCAATGTGCTGGCTATGGATTACTCTGGATTTGATGCTTCTCATACATCTGGCATGTTTTGCATTTTAAAACACTT
CCTTACCACTCTTGGTTATGGAACTTTACAGCTATCTTATATTGATTCTTTGTGCTATTCTAAGCACCATTGGGATGATGAGACCTATCGCCTTGATGGTGGATTGCCTTCAGGATGCTCAGGCACAACAATATTTA
ACACAATTATGAACAACATTGTTGCCCGTGCAGCCGCCGGCTATGCTGCTGAAGGACCTGTTGGCATTCTCTGTTATGGTGATGATATTCTTGTCTCATCTCCTGAGAAATTTCCAGTTTCTGACTGGTTGGAGTTT
TACTCCAGAACACCTTACAAAGTGACTGCAGCTGATAAGAGTGAACAGATTGACTGGCGCGATATCACTCAGTGCACATTCCTTAAACGGGGATTTGTACTGGATGGTAGTCTTGTGCGCCCAGTAATGGATGAACA
ACACTTGGCTGAATTGCTAAAATGGGCTAGACCTGGAACACTACAGGCCAAGTTATTGTCTATTGCCCAGTTGGCTTTTCACCTTCCACGCCAGGCCTACGACCGCCTGATGCTCCCGTTTGAGGAGGCTGGGTATG
AAATACCCTGCCATGAGCGCCTCAATGAGGAGTGGCGTGAAATGTTCACTTGAATAGCTACAGTTGTGAGCAGTTTAGTGATTTTCTGCCTGTTGCCCAAGTAGTTTTAGTTGAGC

Figure S5. Genomic sequence of the rPTV-A Gi2020_1 clone. The infectious molecular clone of PTV-A Gi2020_1 was generated using a pBR322 system. The sequence of parts of the 5'-UTR, the complete ORF, and the 3'-UTR of this strain has been deposited in GenBank under MW824925. The genome of PTV-A Gi2020_1 is AU-rich (55,20%) with 27.8% A, 27.7% U, 21.8% G, and 22.6% C. The individual regions of the genome are annotated and labeled as 5'-UTR (blue), Leader (black), VP4 (sky blue), VP2 (almond), VP3 (green), VP1 (lemon), 2A (orange), 2B (red), 2C (pink), 3A (bold blue), 3B (bold green), 3C (bold red), 3D including stop codon (bold blueberry), 3'-UTR (bold black).
