# Supplementary material for: Completion of the 5′ end of the porcine teschovirus genome reveals a functional S-segment and enables reverse genetics
Source: J Virol. 2026 Aug 11;100(9):e01036-26. doi: 10.1128/jvi.01036-26 (PMC13595913; doi:10.1128/jvi.01036-26)
Supplement: Supplementary file 2 [file jvi.01036-26-s0002.pdf]

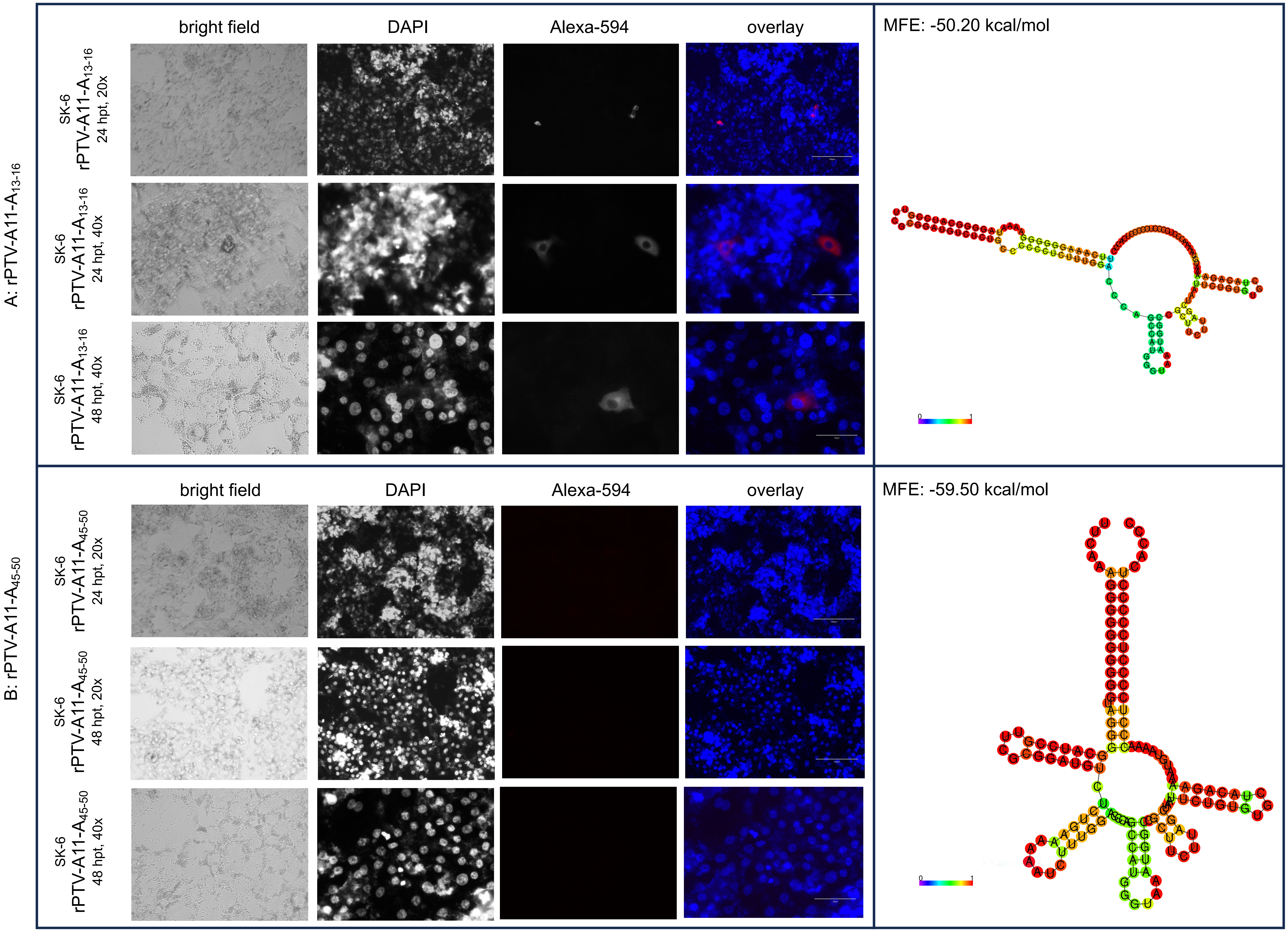

Figure S6. Effect of S-segment mutations on PTV replication and predicted RNA secondary structure. (A) Indirect immunofluorescence analysis of SK-6 cells transfected with rPTV-A11-$A_{13\text{-}16}$ RNA and corresponding RNAfold prediction of the mutated S-segment. At 24 hpt, individual antigen-positive cells displaying the characteristic cytoplasmic staining pattern of PTV infection were detected. Infected cells, but no infection foci were observed at 48 hpt. The predicted RNA structure retained the overall stem-loop architecture of the wild-type S-segment. (B) Indirect immunofluorescence analysis of SK-6 cells transfected with rPTV-A11-$A_{45\text{-}50}$ RNA and corresponding RNAfold prediction of the mutated S-segment. No viral antigen was detected at 24 or 48 hpt. The predicted RNA structure showed a marked rearrangement of the wild-type stem-loop architecture and the formation of multiple short hairpins. Cells were stained with the PTV-specific monoclonal antibody 4B1 and an Alexa Fluor 594-conjugated goat anti-mouse secondary antibody. Nuclei were counterstained with DAPI. Representative overlay images are shown. Scale bars, 75 or 150 µm as indicated with original magnifications of 20x or 40x.

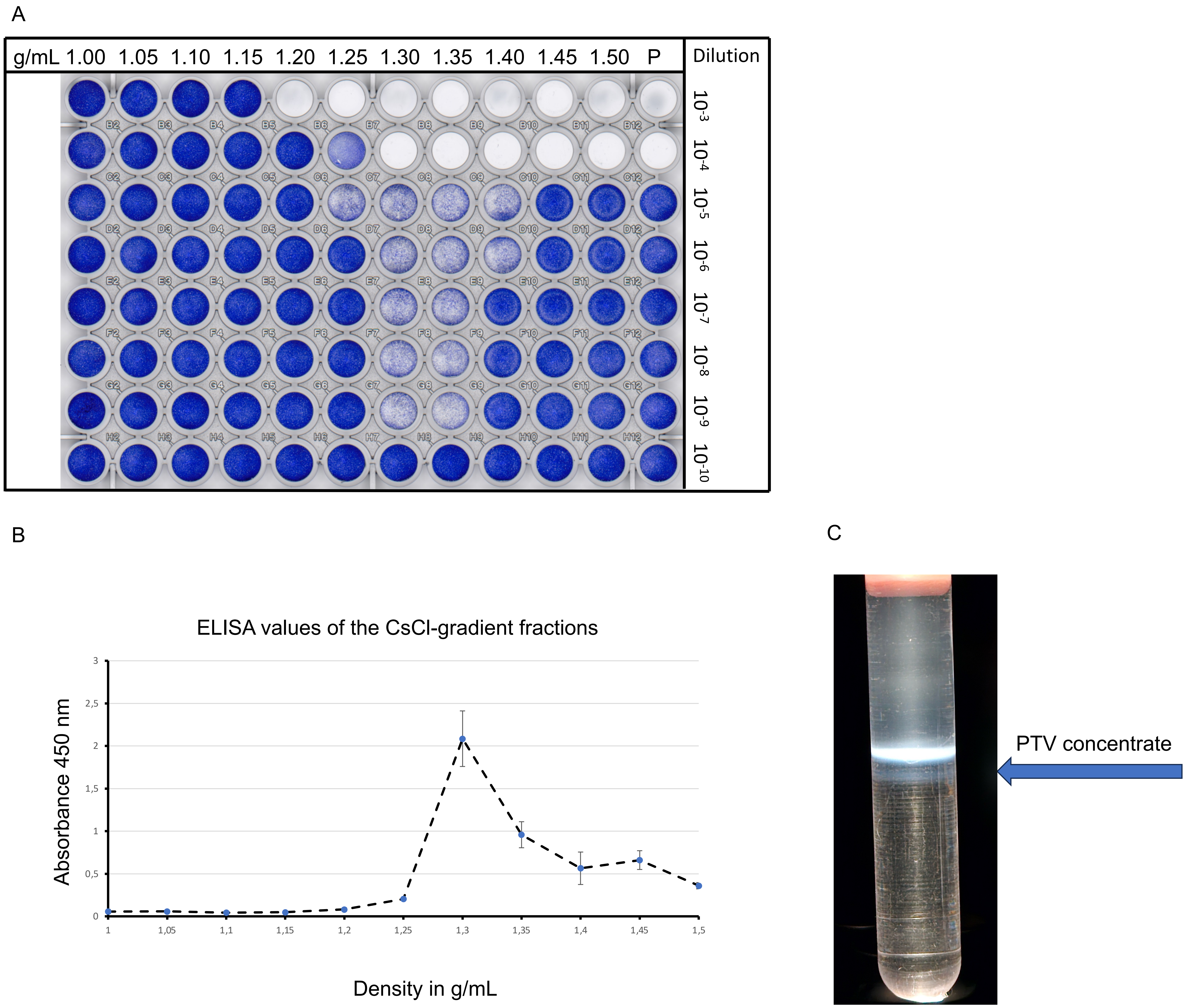

Figure S7. Concentration of PTV-A Gi2020_1 particles by CsCl density gradient centrifugation. (A) Virus particles from 96 mL of infected cell culture supernatant were concentrated by ultracentrifugation and separated according to buoyant density in a continuous CsCl gradient. Twelve fractions with representative densities of 1.0 to 1.5 g/mL and the pellet (P) were selected and titrated in a $TCID_{50}$ assay on SK-6 cells. After 72 hours of incubation, the cells were fixed and stained with crystal violet. Since most of the dead cells were washed away, the toxic effects of CsCl and cytopathic effects due to virus replication can be recognized by the weaker or absent staining. Despite cell damage caused by high concentrations of CsCl in the first dilutions of high-density fractions, high titers of infectious virus were detected in fractions with densities of 1.30 and 1.35 g/mL. (B) Detection of PTV antigen in gradient fractions by ELISA using the PTV-specific monoclonal antibody 4B1. Viral antigen was enriched in the same fractions containing peak infectivity (1.30 and 1.35 g/mL). C) Preparative CsCl step gradient after ultracentrifugation and illumination from below. Virus particles accumulated as a discrete band immediately above the 1.40 g/mL CsCl cushion.
