# Supplementary material for: Completion of the 5′ end of the porcine teschovirus genome reveals a functional S-segment and enables reverse genetics
Source: J Virol. 2026 Aug 11;100(9):e01036-26. doi: 10.1128/jvi.01036-26 (PMC13595913; doi:10.1128/jvi.01036-26)
Supplement: Supplementary file 3 [file jvi.01036-26-s0003.pdf]

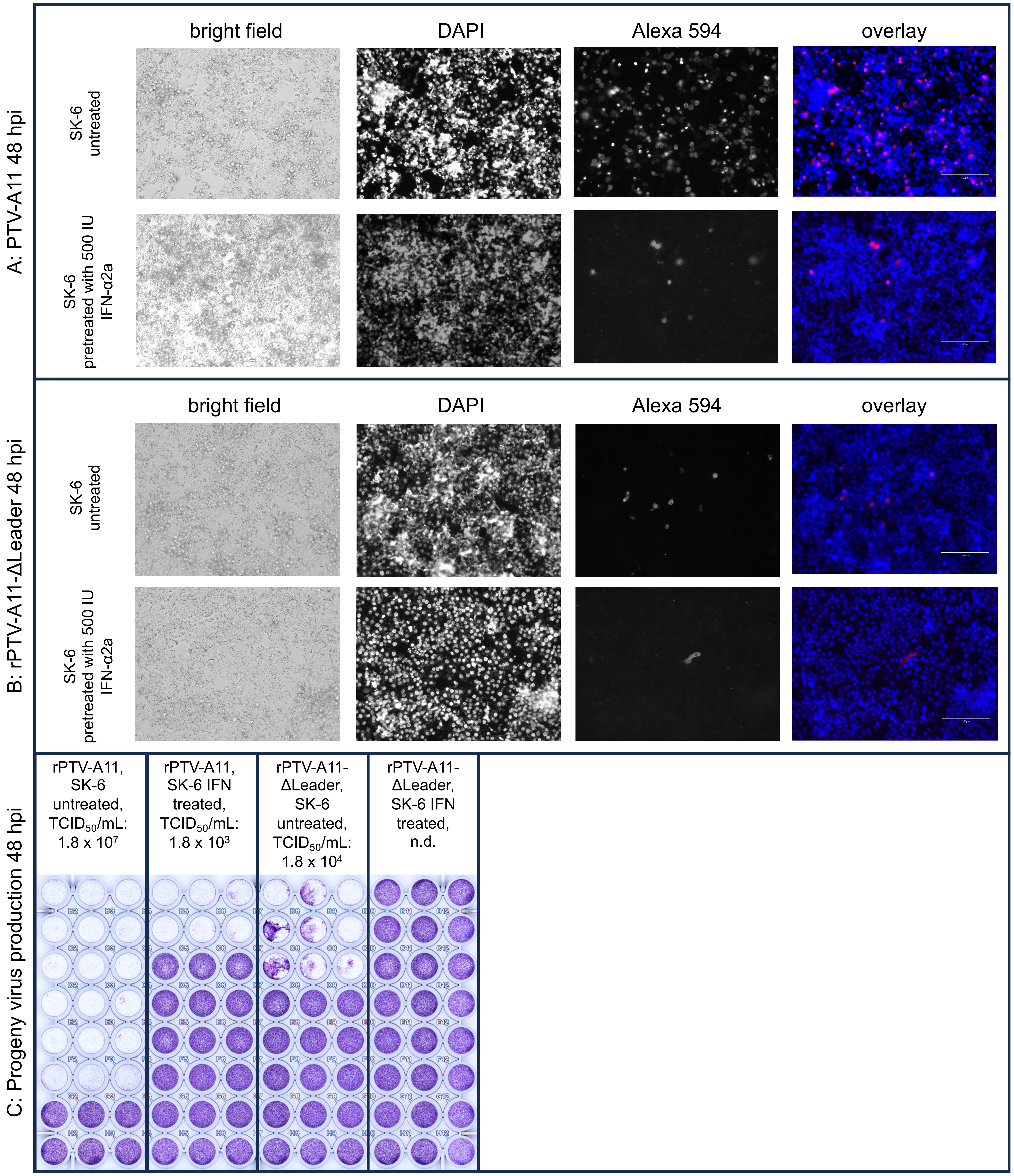

Figure S8. Sensitivity of PTV-A11 and rPTV-A11-ΔLeader to IFN-α2a pretreatment. Indirect immunofluorescence analysis of SK-6 cells pretreated with 500 IU IFN-α2a for 24 h prior to infection with wild-type PTV-A11 or rPTV-A11-ΔLeader. (A) At a MOI of 0.01, wild-type PTV-A11 spread efficiently in untreated cells. Following IFN-α2a pretreatment, infection was markedly reduced and no infection foci were observed, although individual antigen-positive cells remained detectable. (B) At a MOI of 0.01, rPTV-A11-ΔLeader established productive infection in untreated cells but exhibited an attenuated growth phenotype. Following IFN-α2a pretreatment, infection was almost completely abolished, with only a single antigen-positive cell detected. Cells were fixed at 48 hpi and stained with the PTV-specific monoclonal antibody 4B1 followed by an Alexa Fluor 594-conjugated goat anti-mouse secondary antibody. Nuclei were counterstained with DAPI. Representative overlay images are shown. Scale bar, 150 µm with original magnification of 20x. (C) Progeny virus production of PTV-A11 and rPTV-A11-ΔLeader in untreated and IFN-α2a-pretreated SK-6 cells. Cell culture supernatants harvested at 48 hpi were titrated on naïve SK-6 cells, and infectious virus titers were determined by $TCID_{50}$ assay. Owing to its attenuated phenotype, rPTV-A11-ΔLeader produced incomplete monolayer destruction within the 3-day readout period, whereas wild-type PTV-A11 caused complete lysis. No detectable (n.d.) progeny virus production was observed for rPTV-A11-ΔLeader following IFN-α2a pretreatment.

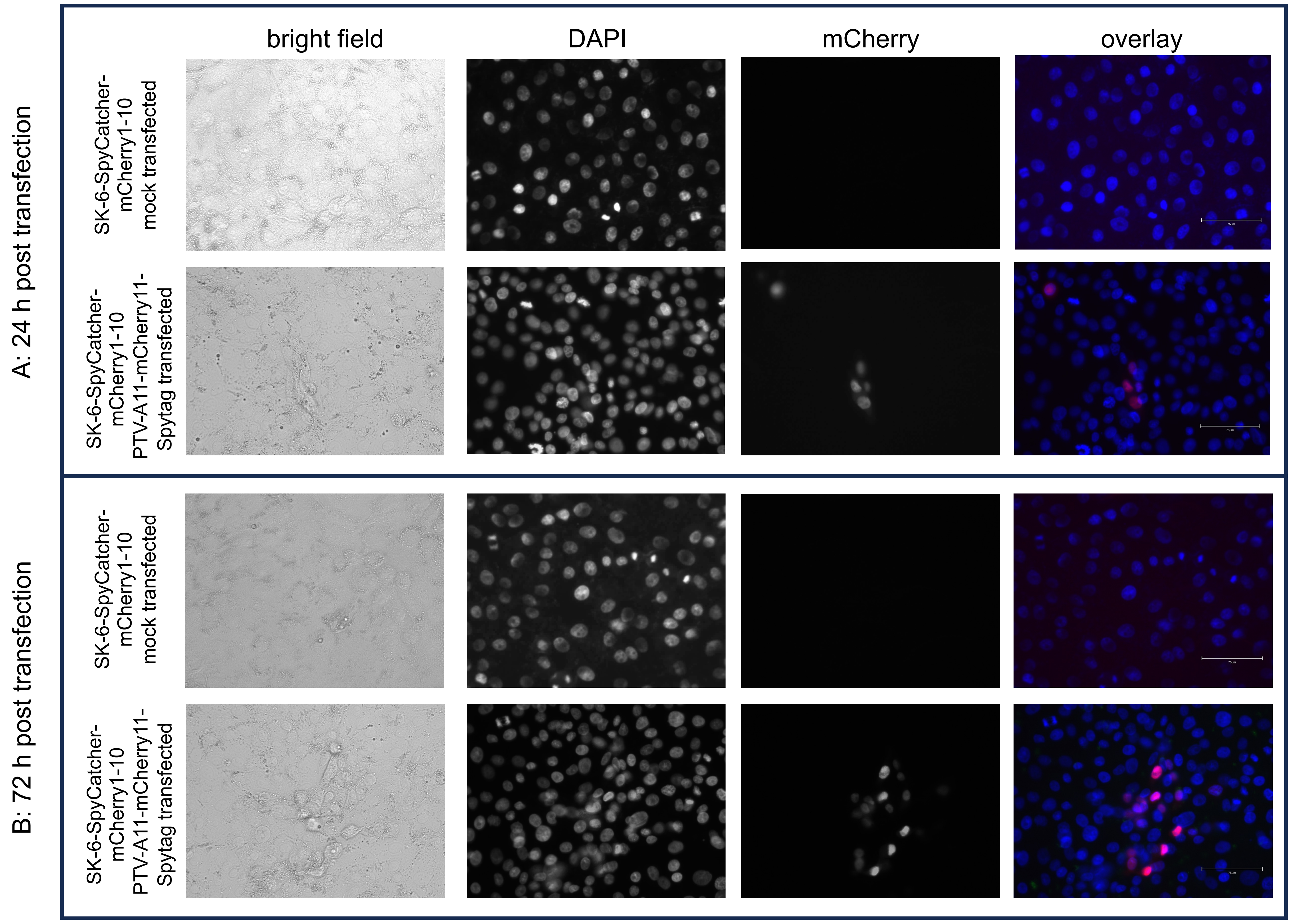

Figure S9. Reconstitution of split-mCherry fluorescence following infection of SK-6-SpyCatcher-mCherry1-10 reporter cells with rPTV-A11-mCherry11-SpyTag. (A) At 24 hpt of rPTV-A11-mCherry11-SpyTag RNA, a nuclear mCherry fluorescence was detected in individual transfected cells, indicating reconstitution of the split-mCherry reporter. (B) Starting at 72 hpt, small fluorescent infection foci were observed, demonstrating slow spread of rPTV-A11-mCherry11-SpyTag to neighboring cells. No fluorescence signal was detected in mock-transfected SK-6-SpyCatcher-mCherry1-10 cells. Nuclei were counterstained with DAPI. Representative overlay images are shown. Scale bar, 75 µm with original magnification of 40x.

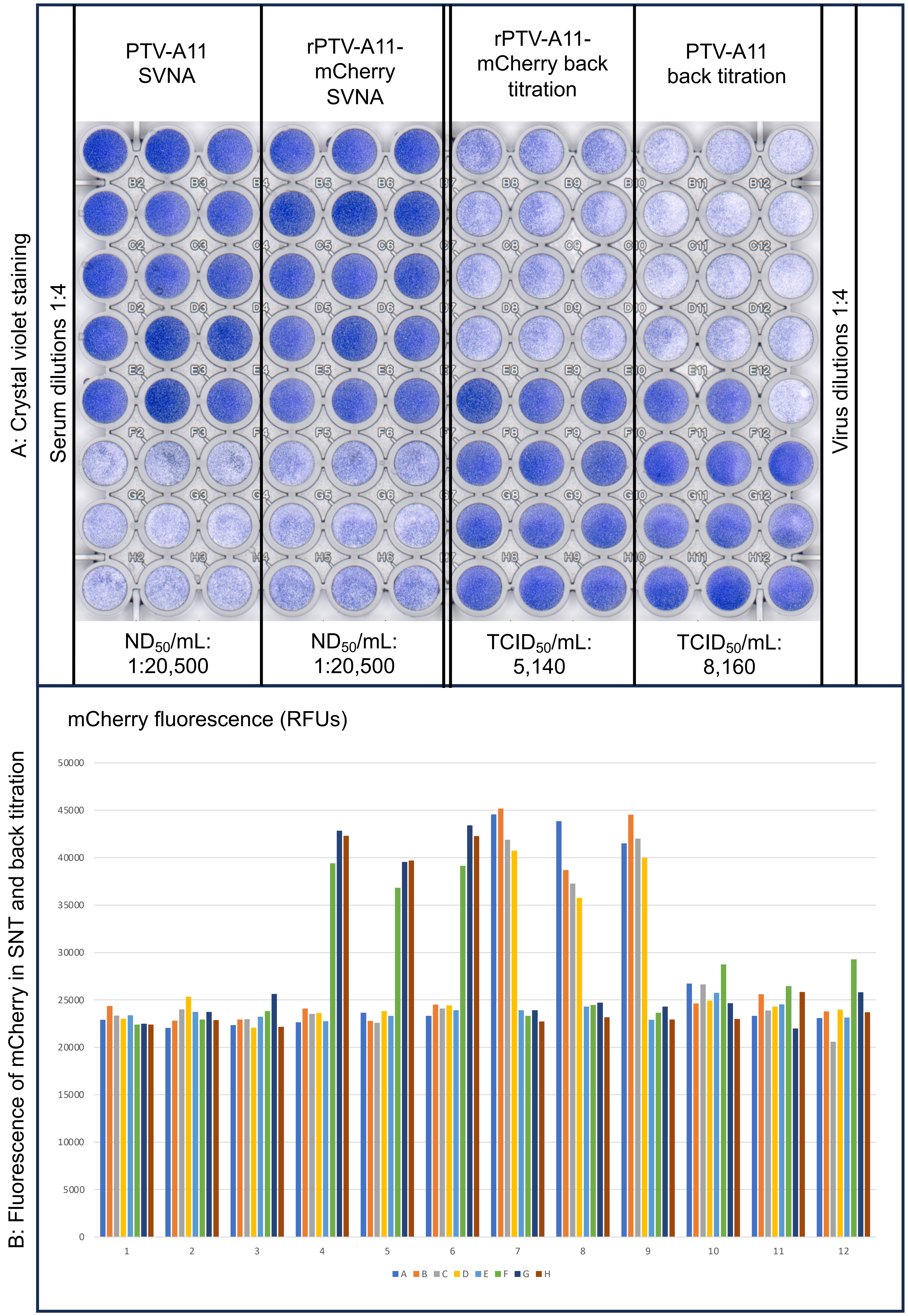

Figure S10. Comparison of serum neutralization assays using PTV-A11 and the reporter virus rPTV-A11-mCherry. (A) Serum virus neutralization assays (SVNAs) performed with wild-type PTV-A11 and the reporter virus rPTV-A11-mCherry using serial fourfold dilutions of a PTV-A11-specific rabbit antiserum. 72 hpi, the cells were fixed and stained with crystal violet. The neutralization titers (ND_{50}/ml) were identical for PTV-A11 and rPTV-A11-mCherry (1:20,500). Concurrent virus back titrations yielded infectious titers of 5.14 x 10^3 $TCID_{50}$/mL for rPTV-A11-mCherry and 8.16 x 10^3 $TCID_{50}$/mL for PTV-A11, confirming comparable starting doses. (B) Quantification of mCherry fluorescence in the same assay plate using a Spark Cyto reader. Fluorescence signals were detected at 48 hpi including the culture medium and are presented as relative fluorescence units (RFU). Wells that exhibited productive infection with rPTV-A11-mCherry showed elevated fluorescence signals, while neutralized or uninfected wells remained at background levels. The fluorescence measurement correlated well with the visual assessment of cytopathic effects at 72 hpi and enabled the automated detection of reporter virus replication at earlier timepoints.
