# Supplementary material for: Completion of the 5′ end of the porcine teschovirus genome reveals a functional S-segment and enables reverse genetics
Source: J Virol. 2026 Aug 11;100(9):e01036-26. doi: 10.1128/jvi.01036-26 (PMC13595913; doi:10.1128/jvi.01036-26)
Supplement: Supplementary file 4 [file jvi.01036-26-s0004.pdf]

Part 2 Supplemental Figures and Legends for „Completion of the 5′ End of the Porcine Teschovirus Genome Reveals a Functional S Segment and Enables Reverse Genetics”

Sebastian Affeldt[1], Sandra Barth[1], Stephanie Schlimmbach[1], Irmin Lobedank[1], Josef Kuehling[2], Thomas Timm[3§], Günter Lochnit[3], Sabrina Becker[2], Enrica Sozzi[4], Sven Reiche[5], Friedemann Weber[1], Gerald Reiner[2], and Benjamin Lamp[1*]

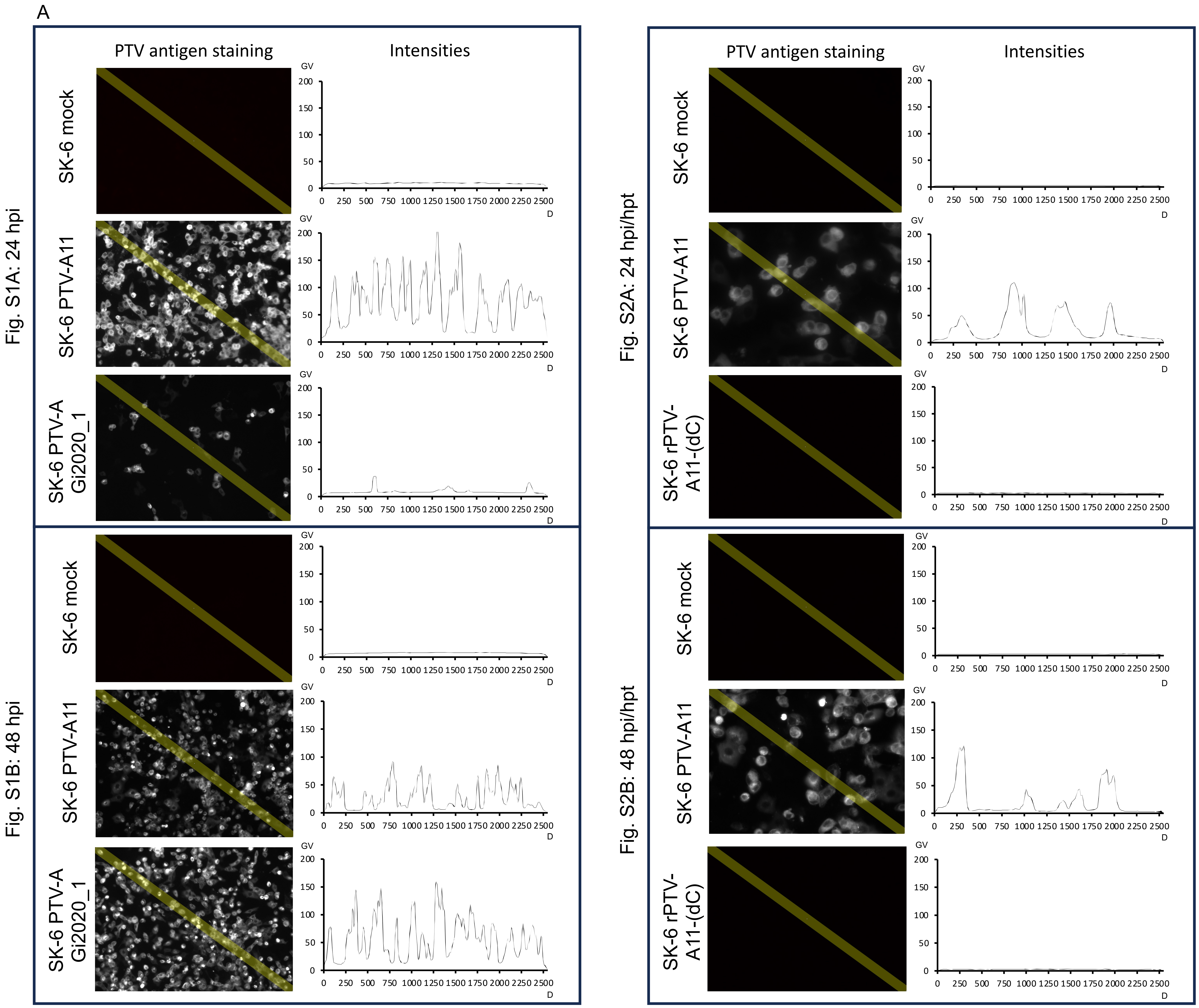

Figure S11. Fluorescence intensity profile analysis of viral antigen and reporter protein expression. (A-E) Images corresponding to the indicated main and supplementary figures are shown together with their respective intensity profiles. For quantitative comparison, original fluorescence images were converted to 8-bit grayscale and intensity was measured along the indicated diagonal region of interest (ROI). Intensity values were extracted using Fiji (ImageJ) and plotted as fluorescence intensity (gray values, GV) versus distance in pixels (D). The resulting profiles illustrate the distribution and intensity of viral antigen staining and reporter fluorescence signals in infected or RNA-transfected cells.

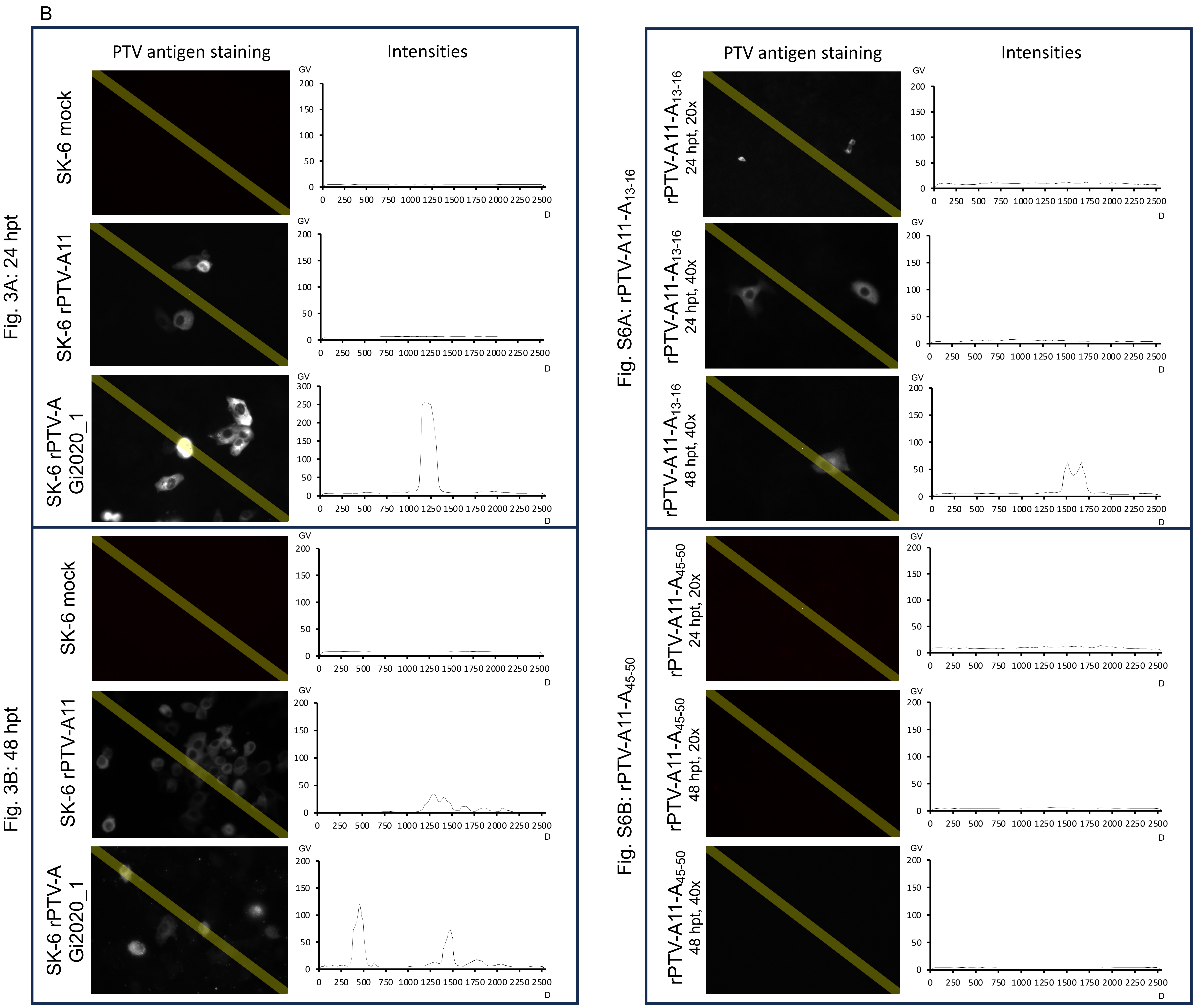
B
PTV antigen staining
Intensities
Fig. 3A: 24 hpt
SK-6 mock
SK-6 rPTV-A11
SK-6 rPTV-A Gi2020_1
Fig. 3B: 48 hpt
SK-6 mock
SK-6 rPTV-A11
SK-6 rPTV-A Gi2020_1
PTV antigen staining
Intensities
Fig. S6A: rPTV-A11-A$_{13-16}$
rPTV-A11-A$_{13-16}$ 24 hpt, 20x
rPTV-A11-A$_{13-16}$ 24 hpt, 40x
rPTV-A11-A$_{13-16}$ 48 hpt, 40x
Fig. S6B: rPTV-A11-A$_{45-50}$
rPTV-A11-A$_{45-50}$ 24 hpt, 20x
rPTV-A11-A$_{45-50}$ 48 hpt, 20x
rPTV-A11-A$_{45-50}$ 48 hpt, 40x
GV
D
0 250 500 750 1000 1250 1500 1750 2000 2250 2500

C

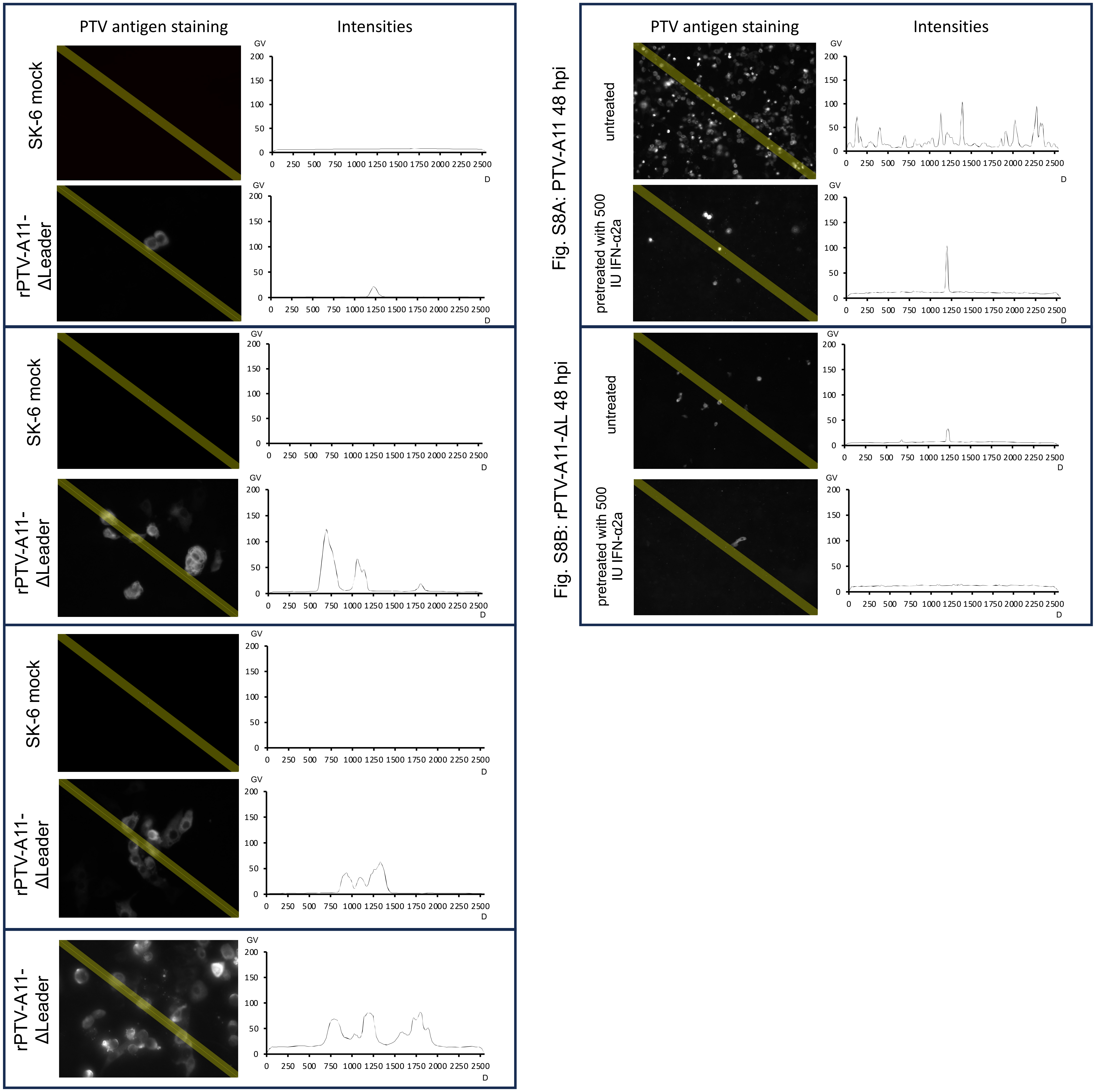

PTV antigen staining
Intensities
Fig. 6A: 24 hpt
SK-6 mock
rPTV-A11-ΔLeader
Fig. 6B: 48 hpt
SK-6 mock
rPTV-A11-ΔLeader
Fig. 6C: 72 hpt
SK-6 mock
rPTV-A11-ΔLeader
Fig. 6D: 24 hpi
rPTV-A11-ΔLeader
Fig. S8A: PTV-A11 48 hpi
untreated
pretreated with 500 IU IFN-α2a
Fig. S8B: rPTV-A11-ΔL 48 hpi
untreated
pretreated with 500 IU IFN-α2a
GV
D
0
50
100
150
200
250
500
750
1000
1250
1500
1750
2000
2250
2500

D

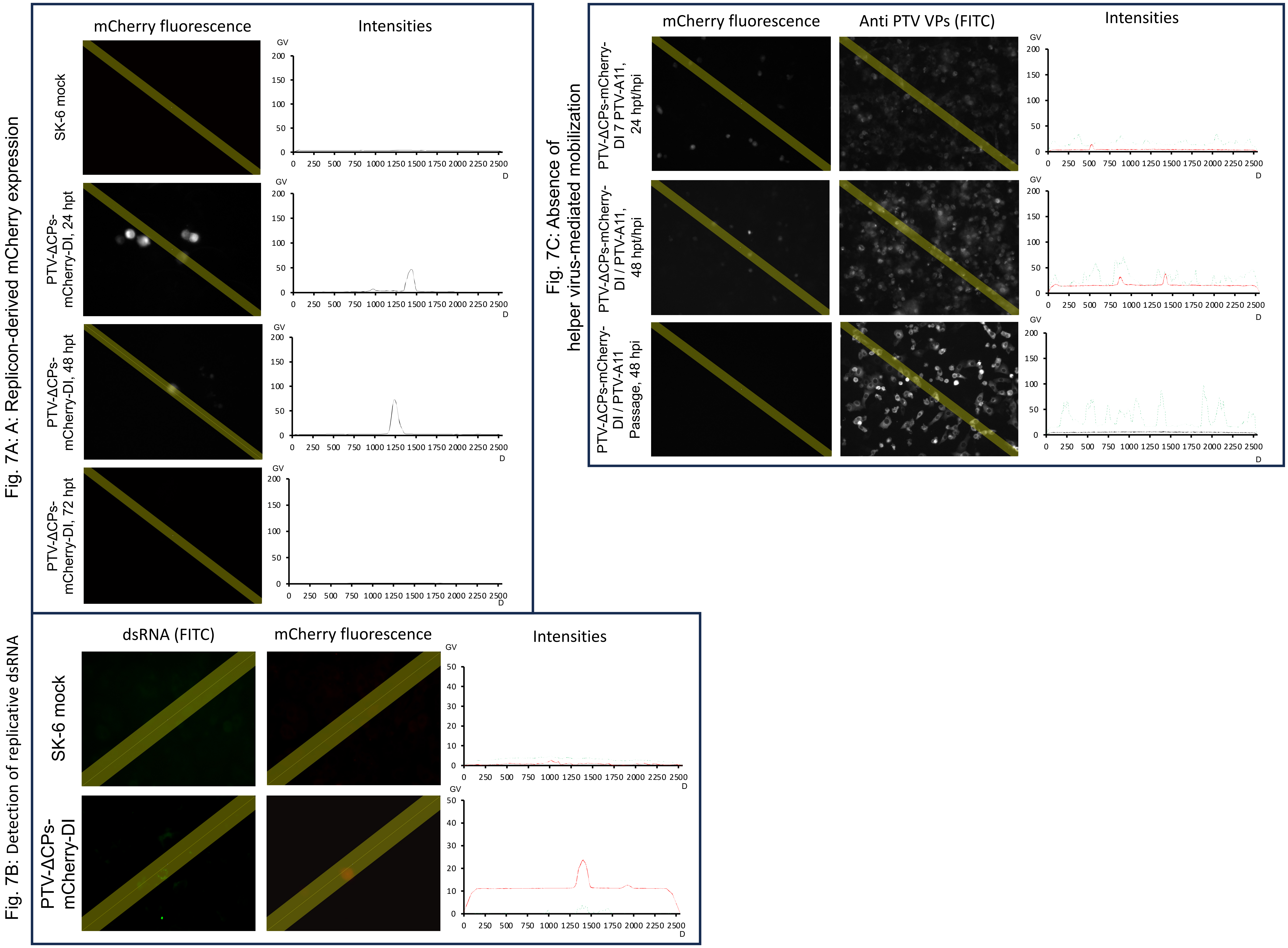
Fig. 7A: A: Replicon-derived mCherry expression
mCherry fluorescence
Intensities
SK-6 mock
PTV-ΔCPs-mCherry-DI, 24 hpt
PTV-ΔCPs-mCherry-DI, 48 hpt
PTV-ΔCPs-mCherry-DI, 72 hpt
GV
D
Fig. 7C: Absence of helper virus-mediated mobilization
mCherry fluorescence
Anti PTV VPs (FITC)
Intensities
PTV-ΔCPs-mCherry-DI 7 PTV-A11, 24 hpt/hpi
PTV-ΔCPs-mCherry-DI / PTV-A11, 48 hpt/hpi
PTV-ΔCPs-mCherry-DI / PTV-A11 Passage, 48 hpi
Fig. 7B: Detection of replicative dsRNA
dsRNA (FITC)
mCherry fluorescence
Intensities
SK-6 mock
PTV-ΔCPs-mCherry-DI

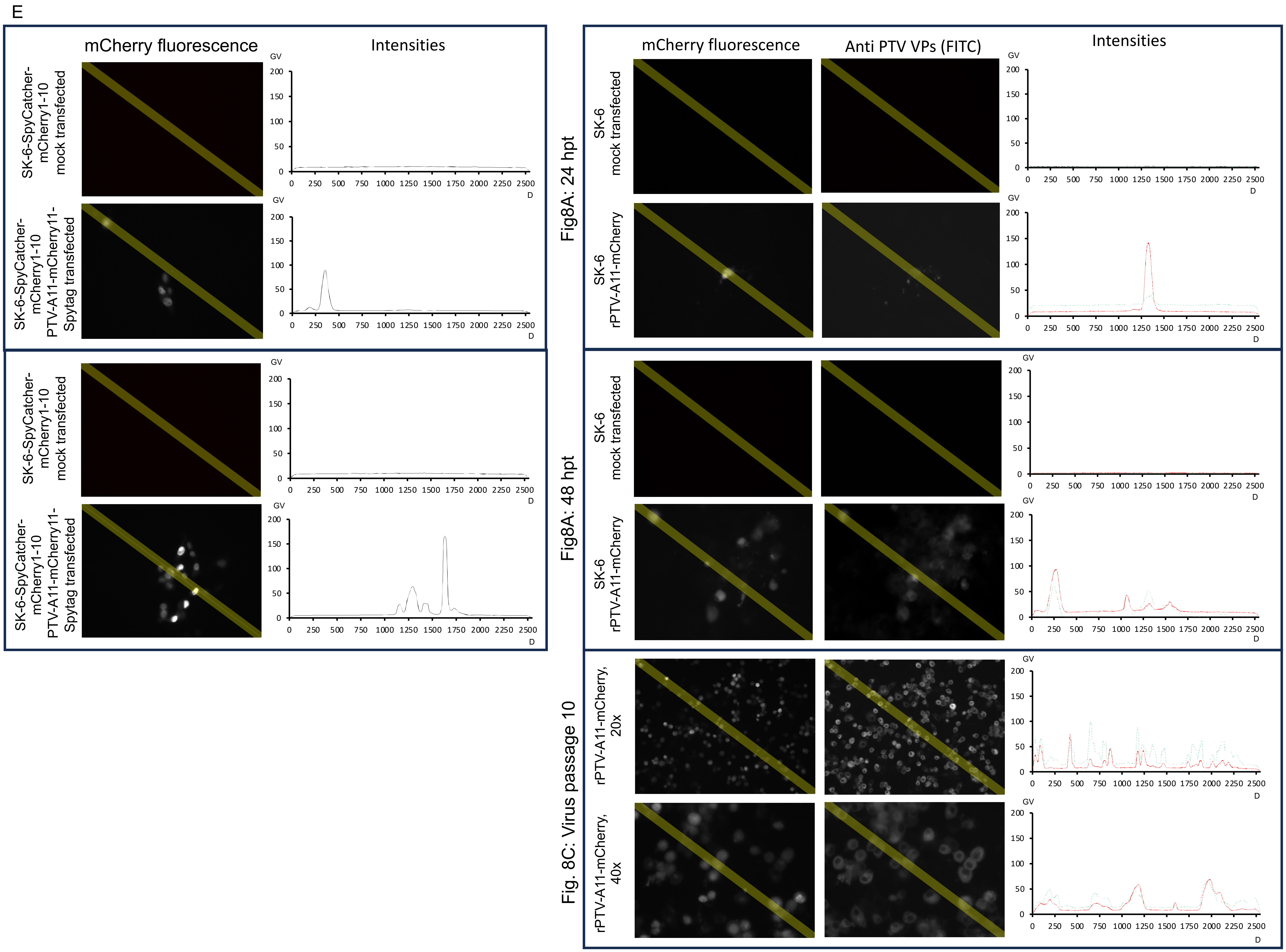
E
mCherry fluorescence
Intensities
Fig. S9A: 24 hpt
SK-6-SpyCatcher-mCherry1-10 mock transfected
SK-6-SpyCatcher-mCherry1-10 PTV-A11-mCherry11-Spytag transfected
Fig. S9B: 72 hpt
SK-6-SpyCatcher-mCherry1-10 mock transfected
SK-6-SpyCatcher-mCherry1-10 PTV-A11-mCherry11-Spytag transfected
mCherry fluorescence
Anti PTV VPs (FITC)
Intensities
Fig8A: 24 hpt
SK-6 mock transfected
SK-6 rPTV-A11-mCherry
Fig8A: 48 hpt
SK-6 mock transfected
SK-6 rPTV-A11-mCherry
Fig. 8C: Virus passage 10
rPTV-A11-mCherry, 20x
rPTV-A11-mCherry, 40x
GV
0
50
100
150
200
D
0
250
500
750
1000
1250
1500
1750
2000
2250
2500
